# Microbial Functional Diversity Correlates with Species Diversity along a Temperature Gradient

**DOI:** 10.1128/msystems.00991-21

**Published:** 2022-02-15

**Authors:** Ilona A. Ruhl, Andriy Sheremet, Angela V. Smirnova, Christine E. Sharp, Stephen E. Grasby, Marc Strous, Peter F. Dunfield

**Affiliations:** a Department of Biological Sciences, University of Calgary, Calgary, Canada; b Department of Geoscience, University of Calgary, Calgary, Canada; c Geological Survey of Canada, Calgary, Canada; MIT

**Keywords:** functional diversity, metabolic diversity, metagenomic diversity, taxonomic diversity, species diversity, temperature, geothermal spring, hot spring

## Abstract

Microbial community diversity is often correlated with physical environmental stresses like acidity, salinity, and temperature. For example, species diversity usually declines with increasing temperature above 20°C. However, few studies have examined whether the genetic functional diversity of community metagenomes varies in a similar way as species diversity along stress gradients. Here, we investigated bacterial communities in thermal spring sediments ranging from 21 to 88°C, representing communities of 330 to 3,800 bacterial and archaeal species based on 16S rRNA gene amplicon analysis. Metagenomes were sequenced, and Pfam abundances were used as a proxy for metagenomic functional diversity. Significant decreases in both species diversity and Pfam diversity were observed with increasing temperatures. The relationship between Pfam diversity and species diversity followed a power function with the steepest slopes in the high-temperature, low-diversity region of the gradient. Species additions to simple thermophilic communities added many new Pfams, while species additions to complex mesophilic communities added relatively fewer new Pfams, indicating that species diversity does not approach saturation as rapidly as Pfam diversity does. Many Pfams appeared to have distinct temperature ceilings of 60 to 80°C. This study suggests that temperature stress limits both taxonomic and functional diversity of microbial communities, but in a quantitatively different manner. Lower functional diversity at higher temperatures is probably due to two factors, including (i) the absence of many enzymes not adapted to thermophilic conditions, and (ii) the fact that high-temperature communities are comprised of fewer species with smaller average genomes and, therefore, contain fewer rare functions.

**IMPORTANCE** Only recently have microbial ecologists begun to assess quantitatively how microbial species diversity correlates with environmental factors like pH, temperature, and salinity. However, still, very few studies have examined how the number of distinct biochemical functions of microbial communities, termed functional diversity, varies with the same environmental factors. Our study examined 18 microbial communities sampled across a wide temperature gradient and found that increasing temperature reduced both species and functional diversity, but in different ways. Initially, functional diversity increased sharply with increasing species diversity but eventually plateaued, following a power function. This pattern has been previously predicted in theoretical models, but our study validates this predicted power function with field metagenomic data. This study also presents a unique overview of the distribution of metabolic functions along a temperature gradient, demonstrating that many functions have temperature “ceilings” above which they are no longer found.

## INTRODUCTION

Ecologists have been documenting the effects of environmental parameters on plant and animal diversity since the mid-1800s (1). One of the strongest correlations is the peak of plant and animal diversity at warm midlatitudes and the diversity decline toward the poles (1). Recently, microbial ecologists have investigated whether diversity gradients can be observed in microbial communities as well. Several abiotic environmental factors have been shown to correlate with microbial species diversity, with decreasing diversity under more extreme conditions of pH (2–5), aridity (6), salinity (7, 8), heavy metals (9), and temperature (4, 10–17).

A relationship between temperature and microbial alpha diversity has been observed in multiple studies of sediments (4, 12), soil (13, 14), microbial mats (10, 11, 17), and ocean water communities (15, 16). Many of these studies have focused on geothermal environments (4, 10–13, 17, 18). These can maintain relatively stable temperatures year-round, even when seasonal or daily air temperature fluctuations are high (19), thereby simplifying correlations with temperature. Studies examining only limited temperature ranges generally find a positive relationship between diversity and temperature at low temperatures (14) and a negative relationship at higher temperatures (10–14, 17, 18). These two trends are consistent with an overall bell-shaped relationship in which diversity peaks at a moderate temperature. Sharp et al. (4) examined geothermal samples covering a temperature range of over 90°C and observed a Gaussian relationship with peak diversity at 24°C (4).

Relatively fewer studies have addressed whether functional diversity (FD) of microbial communities follows similar patterns as species diversity (SD) across gradients of environmental stress. A positive correlation between the two measures is usually assumed, but the nature of this relationship is uncertain. FD may increase linearly with SD, or, alternatively, it may follow an exponential (or power) function, increasing rapidly at low SD and saturating at a relatively low species count (20). The exponential form of the relationship is most commonly observed in studies where community diversity is manipulated and an emergent metabolic process is measured (20). Metagenome-based comparisons of FD versus SD are far less common, but they include two studies of diverse soils where strong positive linear relationships of bacterial species richness and functional gene richness were observed (21, 22). In these studies, species richness was estimated via 16S rRNA gene amplicon sequencing and functional richness via Metagenomics Rapid Annotations using Subsystems Technology (MG-RAST) annotation of raw metagenome reads generated on an Illumina HiSeq. The commonly observed increase in species richness of marine microbial communities with depth has also been correlated with increased metagenomic gene richness in two studies (23, 24), although the exact nature of the FD-SD curve was not clarified. In these studies, functional diversity was estimated by BLAST assignment of raw metagenome reads to KEGG gene categories. Similarly, Louca et al. (25) used a taxon-assigned function approach to show a positive relationship between the number of functional groups of oceanic microbes and species richness. Analysis of a soil gradient in the Tibetan Plateau demonstrated that aridity stress reduced both SD and FD (where FD was based on MG-RAST annotation of metagenome reads), although direct correlation of the two diversity measures was very weak (26).

While there is evidence that temperature limits certain functional properties of bacterial communities (27), no study has quantitatively assessed functional diversity across a wide temperature gradient where dramatic changes in species diversity are apparent. We therefore selected a set of samples spanning from 21.2 to 88.8°C and corroborated the relationship of species diversity to temperature via 16S rRNA gene amplicon sequencing. We then posed the hypothesis that functional diversity would display a positive correlation with species diversity. Metagenomic libraries were constructed from the sediments and translated raw metagenome reads queried against the Pfam database (28) to generate assembly-independent estimates of Pfam diversity, which were used as an index of the functional diversity of each community. We present this Pfam-based approach as an alternative to the BLAST approach primarily used in previous studies. Functional prediction relying on BLAST, usually of short reads, is error prone due to the difficulty in accurate identification. There is also the possibility for systematic biases in BLAST searches against a database biased to mesophilic communities and genomes.

## RESULTS

### Species diversity versus temperature.

**FIG 1:**
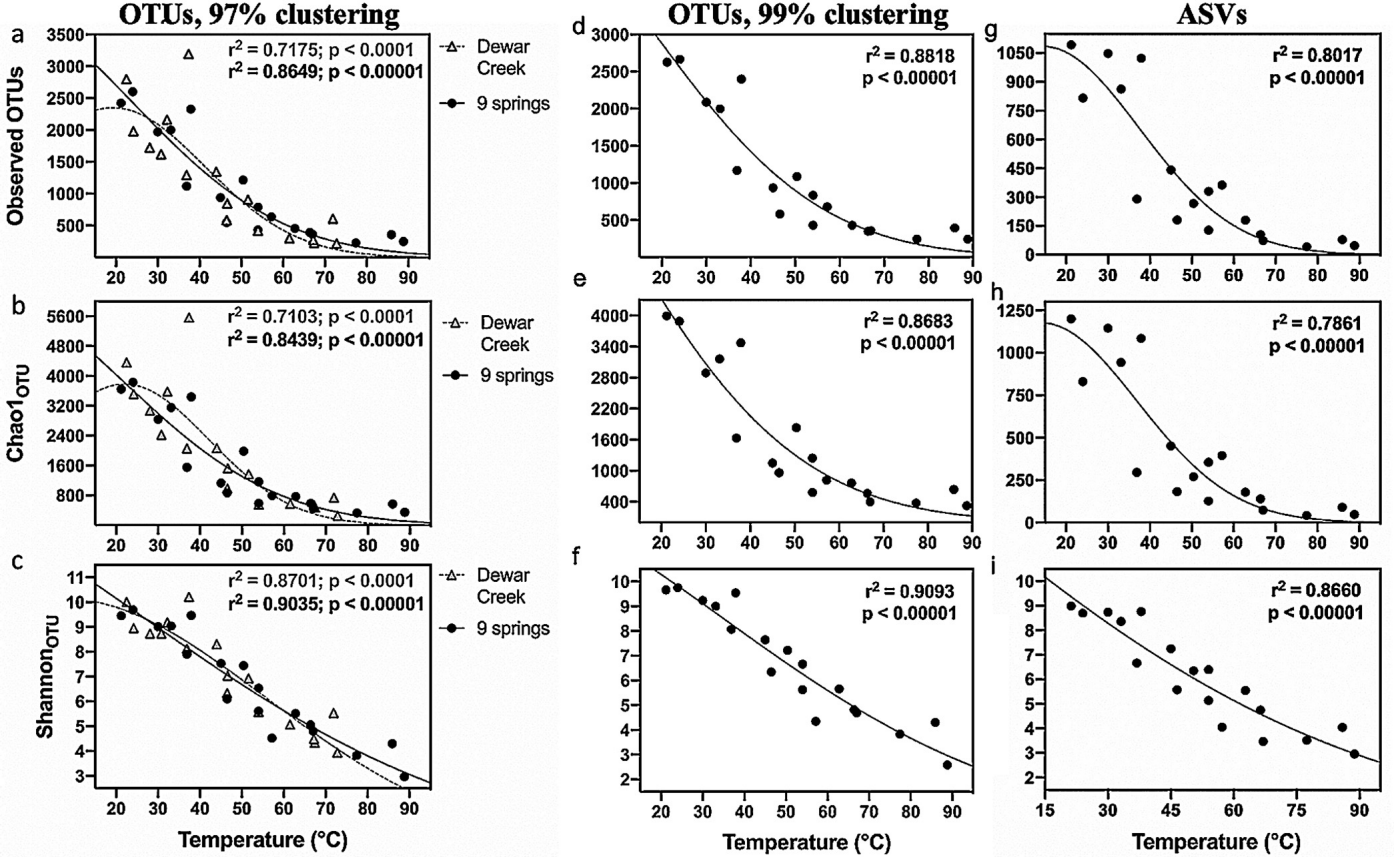
Alpha diversity of *Bacteria* based on 16S rRNA gene amplicon OTUs (a to f) or amplicon sequence variants (ASVs) (g to i) versus temperature in sediments collected from the Dewar Creek hot spring (open triangles) and all 9 springs sampled in this study (black circles). Panels show observed OTUs (a, d, and g), Chao1_OTU_ (b, e, and h), and Shannon_OTU_ (c, f, and i) versus sample temperature. Diversity indices were calculated using 13,460 (a to f) or 8,000 (g to i) 16S rRNA gene sequence reads per sample, amplified with a *Bacteria*-specific primer set, and clustered at 97% (a to c) or 99% (d to i) identity. The best-fit Gaussian least-squares regressions to the Dewar Creek data set (dashed lines, light text) and the 9 spring data set (solid lines, bold text) are shown along with regression fit parameters.

For clarity, species diversity indices calculated from 16S rRNA gene-based operational taxonomic units (OTUs) are designated observed OTUs, Chao1_OTU_, and Shannon_OTU_, whereas functional diversity indices calculated on Pfam data sets are designated observed Pfams, Chao1_Pfam_, and Shannon_Pfam_. The relationship between OTU diversity and temperature was assessed using two data sets, (i) 20 samples collected along the outflow channels of the Dewar Creek hot spring (British Columbia, Canada) (24.2 to 79.8°C), and (ii) 18 samples collected from a set of 9 different pH-neutral geothermal springs (21.2 to 88.8°C) (Table 1) selected from a previous 454 pyrosequencing study (4) and reanalyzed here using Illumina sequencing. For the first data set, sampling was restricted to a single spring to minimize other variables such as pH and water chemistry, and in the second sample set, the pH variability was restricted to 1.25 units (pH 6.85 to 8.10). Observed OTUs, Chao1_OTU_, and Shannon_OTU_ indices calculated from 16S rRNA gene amplicons all showed statistically significant and very strong relationships to temperature for the Dewar Creek data set (*r*^2^ = 0.71 to 0.87; *P* < 0.00001) and for the 9-spring data set (*r*^2^ = 0.84 to 0.90; *P* < 0.00001) (Fig. 1a to c). Nonlinear Gaussian regressions gave the best unbiased fits, although linear regressions were similarly strong (Table 2). Both data sets closely match the relationship reported by Sharp et al. (4), despite the utilization of different PCR primers and a different sequencing platform (Fig. S1).

**TABLE 1:** Description of the geothermal springs examined in this study^*a*^

Hot spring name	Hot spring location	Latitude, longitude	Spring pH range	Sample(s)	Collection yr
Cedar Spring	Canada	54.3565500°N, 128.5422833°W	8.1	HS1	2012
Deer River	Canada	59.5041333°N, 125.9566333°W	7.81	D1	2012
Dewar Creek	Canada	49.9543667°N, 116.5155000°W	7.08–7.93	DC5, DC6, DC8	2010
				DC2	2012
				D37, D47, D54	2015
Fording Mountain	Canada	49.9694500°N, 114.8982833°W	7.20–7.48	FS5	2010
				FS1	2012
Goat Harbour	Canada	53.3569167°N, 128.8881000°W	6.86	GH3	2011
Kiddie Pool Springs	Canada	54.3535500°N, 128.5384500°W	7.79	KP3	2012
Larsen Creek (North)	Canada	60.1987500°N, 125.5127833°W	6.85–7.16	LN4, LN5	2012
Ngatamariki	New Zealand	38.5416667°S, 176.1908333°E	7.31–7.90	N4	2010
				N89	2012
Ram Creek	Canada	50.0327333°N, 115.5929167°W	7.47	RC4	2012

a See Table 3 for individual sample temperatures.

**TABLE 2:** *r*^2^ and *P* values for linear least-squares regressions of data sets presented in this study

Figure panel	Data set	*y* vs x	*r* ^2^	*P* value
1a	Dewar Creek	OTUs 97% vs T	0.6847	0.000041
1a	All springs	OTUs 97% vs T	0.7479	<0.00001
1b	Dewar Creek	Chao1_OTU_ 97% vs T	0.6780	0.000049
1b	All springs	Chao1_OTU_ 97% vs T	0.7240	<0.00001
1c	Dewar Creek	Shannon_OTU_ 97% vs T	0.8626	<0.00001
1c	All springs	Shannon_OTU_ 97% vs T	0.8915	<0.00001
1d	All springs	OTUs 99% vs T	0.7491	<0.00001
1e	All springs	Chao1_OTU_ 99% vs T	0.7324	<0.00001
1f	All springs	Shannon_OTU_ 99% vs T	0.8981	<0.00001
1g	All springs	ASVs vs T	0.6962	0.000017
1h	All springs	Chao1_ASV_ vs T	0.6783	0.000027
1i	All springs	Shannon_ASV_ vs T	0.8484	<0.00001
2a	All springs	Pfams vs T	0.6255	0.000093
2b	All springs	Chao1_Pfam_ vs T	0.5316	0.000597
2c	All springs	Shannon_Pfam_ vs T	0.6089	0.000133
3a	All springs	Pfams vs OTUs	0.4576	0.00205
3b	All springs	Chao1_Pfams_ vs Chao1_OTUs_	0.3811	0.006346
3c	All springs	Shannon_Pfams_ vs Shannon_OTUs_	0.6083	0.000135
3d	All springs	Pfams vs ASVs	0.4224	0.003506
3e	All springs	Chao1_Pfams_ vs Chao1_ASVs_	0.3492	0.009814
3f	All springs	Shannon_Pfams_ vs Shannon_ASVs_	0.6480	0.000056
SF2a	All springs	Pfams vs OTUs	0.7216	0.000016
SF2b	All springs	Chao1_Pfams_ vs Chao1_OTUs_	0.6547	0.000084
SF2c	All springs	Shannon_Pfams_ vs Shannon_OTUs_	0.6414	0.000112
SF2d	All springs	Signature_Pfams vs OTUs	0.4531	0.002202
SF2e	All springs	Chao1_Signature_Pfams_ vs Chao1_OTUs_	0.3920	0.005441
SF2f	All springs	Shannon_Signature_Pfams_ vs Shannon_OTUs_	0.5862	0.000213
SF5a	Power et al	OTUs vs T	0.2179	<0.00001

10.1128/mSystems.00991-21.1 FIG S1 Shannon_OTU_ diversity of *Bacteria* based on 16S rRNA gene amplicon OTUs versus temperature in sediments collected along three temperature transects from the Dewar Creek hot spring (dark gray triangles) and from 18 other geothermal sediments collected from a total of 9 springs (black circles) overlaid on the dataset of Shannon_OTU_ diversity of bacterial and archaeal communities reported by Sharp et al. (4), shown in light gray. The Shannon diversity index was calculated using 13,460 16S rRNA gene sequence reads per sediment sample, amplified with a *Bacteria*-specific primer set, and clustered at 97% identity. The solid black line and the dashed gray line represent the best-fit nonlinear Gaussian least-squares regressions to the data. Figure from Sharp et al. (4), is reprinted with permission from Springer Nature, The ISME Journal (Humboldt’s spa: microbial diversity is controlled by temperature in geothermal environments, Sharp *et al*., 2014). Download FIG S1, PDF file, 0.3 MB. Copyright © 2022 Ruhl et al. 2022 Ruhl et al. https://creativecommons.org/licenses/by/4.0/ This content is distributed under the terms of the Creative Commons Attribution 4.0 International license.

**FIG 2:**
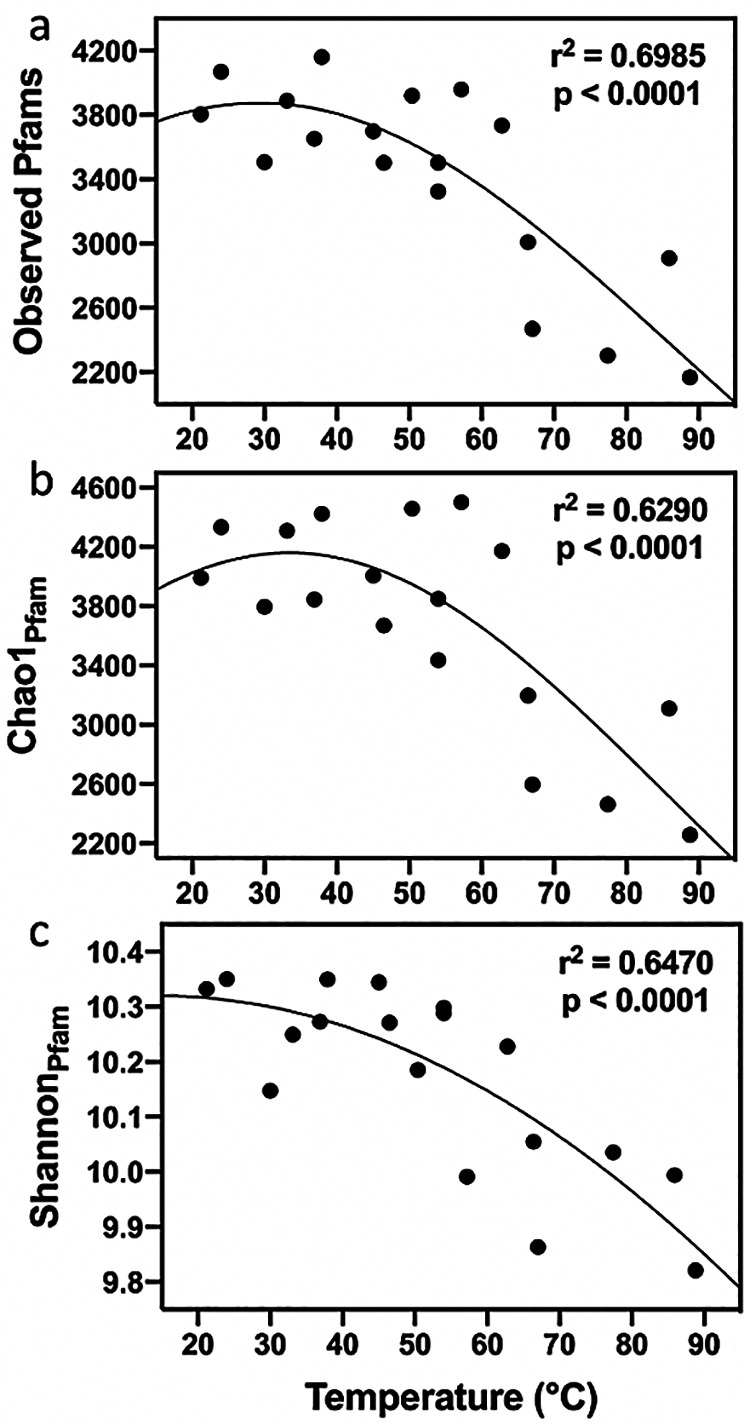
Alpha diversity indices calculated on Pfams detected in metagenomes of geothermal springs. Panels show observed Pfams (a), Chao1_Pfam_ (b), and Shannon_Pfam_ (c) versus sample temperature. Diversity indices were calculated using 600,000 Pfam-assigned reads per sample. The solid lines represent the best-fit nonlinear Gaussian least-squares regressions to the data.

10.1128/mSystems.00991-21.2 FIG S2 Alpha diversity indices calculated on Pfam-assigned reads detected in bacterial communities of geothermal springs after an extra validation step was applied (as described in Fig. S6) (a to c) and 56 Pfams associated with signature biogeochemical pathways (listed in Table S1) (d to f) detected in bacterial communities of geothermal springs. Panels show observed Pfams (a), Chao1_Pfam_ (b), Shannon_Pfam_ (c), observed signature Pfams (d), Chao1_signature_Pfam_ (e), and Shannon_signature_Pfam_ (f) versus sample temperature. For each geothermal spring sample, diversity indices were calculated using either 700,000 validated Pfams (a to c) or 600,000 Pfam-assigned reads, which were subsequently filtered to include only signature Pfams (d to f) per sample. The solid lines represent the best-fit nonlinear Gaussian least-squares regressions to the data. Results are similar to those of unvalidated Pfam-assigned reads (Fig. 2). Download FIG S2, PDF file, 0.4 MB. Copyright © 2022 Ruhl et al. 2022 Ruhl et al. https://creativecommons.org/licenses/by/4.0/ This content is distributed under the terms of the Creative Commons Attribution 4.0 International license.

10.1128/mSystems.00991-21.4 FIG S4 Average number of unique Pfams in bacterial and archaeal genomes, depending on their temperature preferences. Classes are HT, hyperthermophiles (*T*_op_ > 80°C; *n* = 67); T, thermophiles (60°C < *T*_opt_ < 80°C; *n* = 166); TM, moderate thermophiles (45°C <*T*_opt_ <60°C; *n* = 142); all thermophiles (*T*_opt_ > 45°C; *n* = 377); all genomes, the entire dataset of 2,363 bacterial genomes described in Sheremet et al. (30), comprised of >85% mesophiles. Error bars represent +1 SEM. The database was created by first parsing the metadata for all genomes in the IMG (Integrated Microbial Genomes) database for the term “thermophile.” The optimum temperatures for each genome representing a validated species were then manually verified using the metadata in the BacDive website (https://bacdive.dsmz.de). Entries for uncultured microbes were assumed to have an optimum temperature equal to the sampling temperature. All duplicate species and any entries with uncertain metadata were eliminated. Download FIG S4, PDF file, 0.1 MB. Copyright © 2022 Ruhl et al. 2022 Ruhl et al. https://creativecommons.org/licenses/by/4.0/ This content is distributed under the terms of the Creative Commons Attribution 4.0 International license.

10.1128/mSystems.00991-21.6 FIG S6 Flowchart summarizing the assignment and validation of Pfams to metagenomic raw reads. Pfam assignments were validated by cross-referencing them to the Pfam assignments made (also via HMMER) to amino acid sequences of GenomeDatabase. To perform the validation, the translated metagenome reads that were assigned a Pfam were first extracted from the complete translated metagenome dataset using the subseq program of the seqtk package (https://github.com/lh3/seqtk). Second, the extracted reads were aligned against the GenomeDatabase amino acid sequences using DIAMOND (69). As before, the best GenomeDatabase entry hit for each translated metagenome read was selected from the DIAMOND output data using R. Third, the GenomeDatabase entries that most closely aligned with a metagenome read were extracted from the HMMER output of the alignment between the GenomeDatabase and the Pfam database using R. This resulted in a final dataset that contained a metagenome read, the Pfam associated with that read, the GenomeDatabase entry that that metagenome read most closely aligned to, and the Pfam associated with that GenomeDatabase entry. Lastly, the two Pfam assignments were compared using R; a Pfam assignment to a metagenomic read was considered to be validated when it matched the Pfam assignment made to the GenomeDatabase entry that the read aligned with. Download FIG S6, PDF file, 0.4 MB. Copyright © 2022 Ruhl et al. 2022 Ruhl et al. https://creativecommons.org/licenses/by/4.0/ This content is distributed under the terms of the Creative Commons Attribution 4.0 International license.

The temperature-diversity relationship was consistent across multiple levels of OTU clustering (Fig. 1a to f), and OTU analyses were consistent with analyses using individual amplicon sequence variants (ASVs) (Fig. 1g to i). Fewer total ASVs were estimated in the sample set than OTUs because of more stringent filtering. However, the fundamental shape of the temperature-diversity relationship was not greatly changed by using ASVs instead of OTUs.

### Pfam diversity versus temperature.

The 18 metagenomic libraries totaled 61.48 Gb of data and 182,225,430 total reads, with 5 to 26 million reads per sample (Table 3). The percentage of raw reads that could be assigned to a Pfam ranged from 14.9 to 31.7 and showed no relationship to temperature (*r*^2^ = 0.0586; *P* = 0.3333; data not shown). Rarefaction curves of total Pfams began to level off after about 600,000 Pfam-assigned reads, suggesting that sampling additional reads above 600,000 introduced a relatively small number of new Pfams into the data set (data not shown). Similar results were obtained from rarefaction curves for Chao1_Pfam_ and Shannon_Pfam_ (data not shown). Therefore, each sample was rarefied to 600,000 Pfam-assigned reads. Regressions were performed on different alpha diversity indices calculated from the Pfam data versus sample temperature. Nonlinear Gaussian regressions gave the best unbiased fits (Fig. 2), although linear regressions were also significant (Table 2). Observed Pfams, Chao1_Pfam_, and the Shannon_Pfam_ index all showed statistically significant relationships to temperature (*r*^2^ = 0.63 to 0.70; *P* < 0.0001). The strong relationships held after an extra validation step of the Pfam data set using BLAST (Fig. S2a to c in the supplemental material) (*r*^2^ = 0.71 to 0.78; *P* < 0.0001).

**TABLE 3:** Measurement of each sample’s *in situ* temperature and properties of the metagenomes constructed from each sample analyzed in this study

Sample	Temp (°C)	Avg. library size (bp)	Library size calculation method	MiSeq reagent kit	Gb	Total no. of reads (F + R)^*a*^	No. of reads with assigned Pfam	% reads with assigned Pfam
FS1	21.2	890	Bioanalyzer	v3, 600 cycles	0.85	2,660,450	632,763	23.8
FS5	24.0	1,065	Bioanalyzer	v3, 600 cycles	1.65	6,999,658	1,043,427	14.9
D1	30.0	1,120	Bioanalyzer	v3, 600 cycles	3.3	10,330,470	2,405,195	23.3
LN4	33.1	1,423	Bioanalyzer	v3, 600 cycles	5.13	16,457,856	3,412,953	20.7
D37	36.9	753	TapeStation	v3, 600 cycles	2.66	5,173,070	947,243	18.3
RC4	37.9	1,017	Bioanalyzer	v3, 600 cycles	1.22	3,840,888	886,841	23.1
N4	45.0	1,128	Bioanalyzer	v3, 600 cycles	6.08	19,154,944	5,093,693	26.6
D47	46.5	642	TapeStation	v3, 600 cycles	2.9	5,824,428	1,014,183	17.4
LN5	50.4	1,040	Bioanalyzer	v3, 600 cycles	8.25	26,554,712	6,802,478	25.6
KP3	54.0	1,122	Bioanalyzer	v3, 600 cycles	3.91	5,260,764	936,895	17.8
D54	54.0	617	TapeStation	v3, 600 cycles	2.57	12,313,914	3,260,884	26.5
GH3	57.2	1,689	Bioanalyzer	v3, 600 cycles	1.97	6,182,300	1,473,623	23.8
DC8	62.8	1,409	Bioanalyzer	v3 600 cycles	5.22	16,110,352	3,478,277	21.6
DC2	66.4	1,259	Bioanalyzer	v2, 500 cycles	4.12	15,015,840	3,326,274	22.2
HS1	67.0	700	TapeStation	v3, 600 cycles	3.42	7,037,502	1,289,748	18.3
DC6	77.4	1,365	Bioanalyzer	v3, 600 cycles	2.48	7,842,250	2,486,868	31.7
DC5	85.9	1,414	Bioanalyzer	v3, 600 cycles	3.13	9,873,004	2,613,435	26.5
N89	88.8	649	TapeStation	v3, 600 cycles	2.62	5,593,028	982,600	17.6

a F = forward reads; R = reverse reads.

### Signature Pfams.

Each metagenome was also screened for the presence or absence of 56 “signature Pfams” generated from RDP’s FunGene repository (Table S1). A total of 51 were detected. Linear regressions of observed signature Pfams, Chao1_signature___Pfam_, and Shannon_signature___Pfam_ versus temperature (Fig. S2d to f) were all statistically significant (*r*^2^ = 0.44 to 0.62; *P* < 0.01). This reduced signature Pfam data set, therefore, followed a similar pattern as the overall Pfam data set.

10.1128/mSystems.00991-21.7 TABLE S1 List of 56 Pfams used in the signature Pfam analysis and the description of each Pfam. The Pfam description is the name of each corresponding Pfam in version 31.0 of the Pfam database. Pfams were generated using the list of genes associated with biogeochemical cycles provided by RDP’s FunGene repository (65). The FunGene column lists these genes. Download Table S1, PDF file, 0.04 MB. Copyright © 2022 Ruhl et al. 2022 Ruhl et al. https://creativecommons.org/licenses/by/4.0/ This content is distributed under the terms of the Creative Commons Attribution 4.0 International license.

### Pfam diversity versus species diversity.

**FIG 3:**
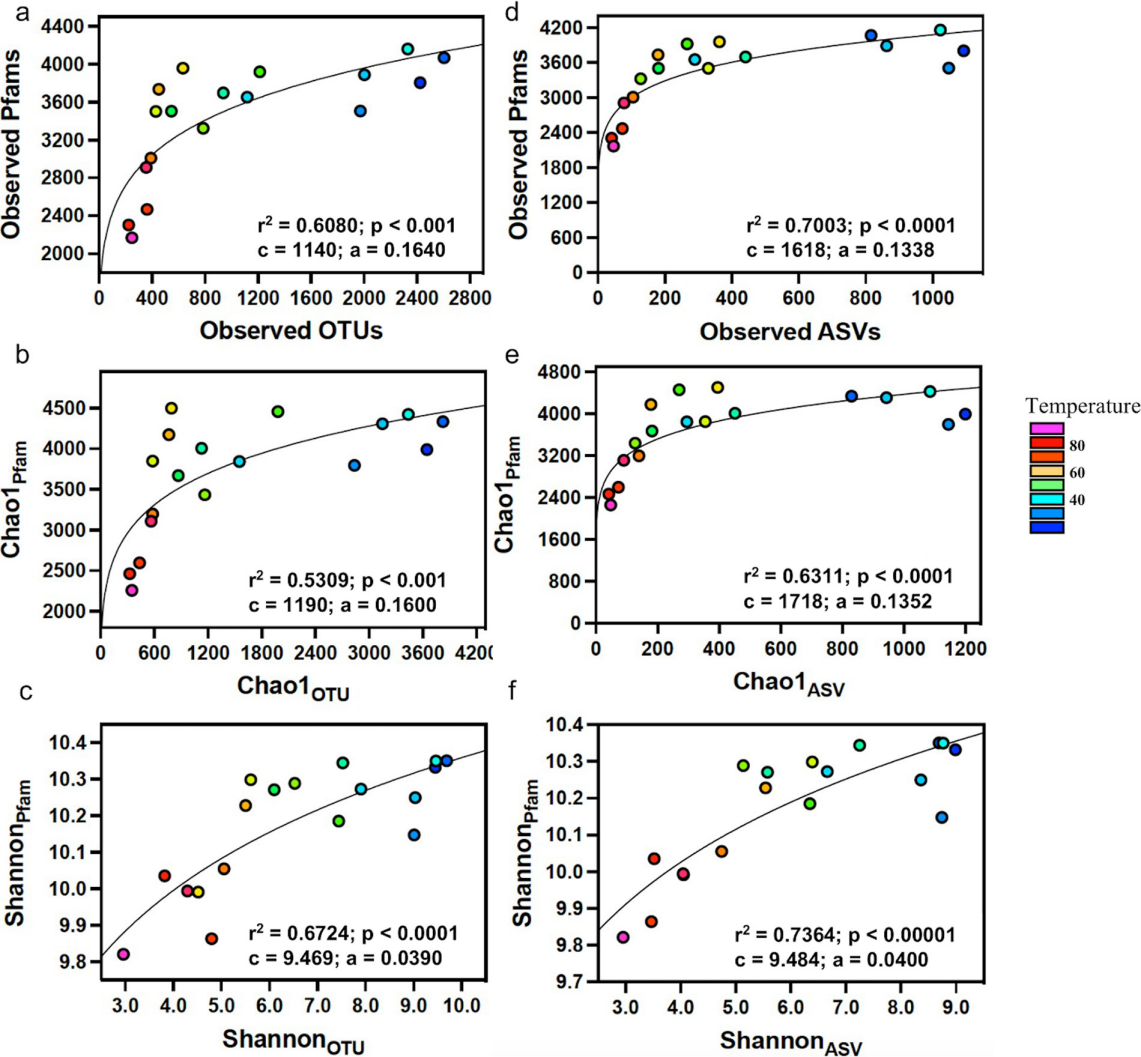
Functional diversity of *Bacteria* based on Pfam analysis versus taxonomic diversity based on 16S rRNA gene amplicon OTU (a to c) or ASV (d to f) analysis in geothermal spring sediments. Panels show observed occurrences (a and d), Chao1 (b and e), and Shannon (c and f). The solid lines represent the best-fit power (FD = *c*SD^a^) regressions to the data where FD is functional diversity (y axis) and SD is species diversity (x axis).; *c* and *a* indicate the best-fit values to the model. Colors of data points correspond to temperature in degrees Celsius.

Comparing the plots of species (OTU) diversity versus temperature (Fig. 1) to plots of Pfam diversity versus temperature (Fig. 2) reveals some distinct features. First, the absolute range of Pfam variability is smaller, suggesting that the reduction of Pfams with increasing temperature was slower than the reduction in OTUs. Second, while the OTU curves are steepest at moderate temperatures (<60°C), the Pfam curves are steepest at high temperatures (>60°C). Diversity metrics for OTUs versus Pfams were therefore compared more closely. Regressions of Pfam diversity versus OTU or ASV diversity fit well to a power model (Fig. 3) (OTU, *r*^2^ = 0.56 to 0.67, *P* < 0.001; ASV, *r*^2^ = 0.63 to 0.74, *P* < 0.0001), indicating that Pfam diversity increased only hypometrically with OTU diversity. That is, Pfam diversity changed rapidly with changing OTU diversity across the low-diversity thermophilic communities (60 to 90°C), but Pfam diversity changed much more slowly across mesophilic communities (below 60°C). Above a Chao_OTU_ of about 800, Pfams stayed nearly constant as more OTUs were added (Fig. 3).

Metagenome diversity as a power function of species diversity has been reported based on *in silico* study. Miki et al. (29) constructed orthologue accumulation curves by sampling from a pool of microbial genomes and concluded that orthologue richness (multifunctionality [MF]) was a power function of species richness (SR). They proposed that in the power model MF = *c*SR^a^, component *a* is a measure of community “multifunctional redundancy.” However, their estimates of *a* of 0.55 to 0.75 were based on orthologues and hence are not directly comparable to Pfams. We therefore reproduced a similar experiment to Miki et al. (29), using a reference data set containing Pfam profiles of 2,363 bacterial genomes as described in Sheremet et al. (30). Randomized collectors curves of Pfam accumulation with sequential random genome sampling fit reasonably well to the power function MF = *c*SR^a^, with *c* equal to 5,351 and *a* equal to 0.0770 (95% confidence interval [CI], 0.0769 to 0.0771) (Fig. S3a). For our experimental field data (Fig. 3), the same power function fit to Chao1_Pfam_ (MR) versus Chao1_OTU_ (SR) produced *c* of 1,190 and *a* of 0.164 (95% CI, 0.0775 to 0.242).

10.1128/mSystems.00991-21.3 FIG S3 (a) Pfam accumulation curves from a sample set of 2,363 bacterial genomes described in Sheremet et al. (30). The genomes were sampled in sequential steps of 10 genomes. This was repeated 100 times and averages plotted. The data were fit to a power function [Pfams = *c*(Genomes)^a^] using the Statistical Package for the Social Sciences (SPSS). The power function was seen to be an excellent fit at high numbers of genomes sampled (>50) but was very biased at lower genome samplings. Therefore, only samplings of >300 genomes are plotted, as this roughly matched the lowest estimated species diversity in the field samples (327). The curve fit gave parameters of *c* of 5,351 and *a* of 0.0770 (95% CI, 0.0769 to 0.0771). (b) Pfam accumulation curves in a simulated set of genomes comprising only thermophiles and hyperthermophiles (optimal temperature [*T*_opt_] > 60°C) in low-diversity communities (30 to 237 genomes) and a subset of all bacteria (mostly mesophiles [30]) in higher-diversity communities (>1,000 genomes), which simulates the situation in our field samples. Not enough moderate thermophile genomes were available to include communities of 237 to 1,000 genomes. Each curve (thermophiles or full genomes) fit well alone to a power function (thermophiles and hyperthermophiles, *c* = 2,566, *a* = 0.159; all genomes, *c* = 5,330, *a* = 0.078). However, trying to force a power function through a combination of the two resulted in a much higher multifunctional redundancy *a* (*c* = 2,117, *a* = 0.201). Download FIG S3, PDF file, 0.05 MB. Copyright © 2022 Ruhl et al. 2022 Ruhl et al. https://creativecommons.org/licenses/by/4.0/ This content is distributed under the terms of the Creative Commons Attribution 4.0 International license.

### Beta diversity of Pfams versus temperature.

**FIG 4:**
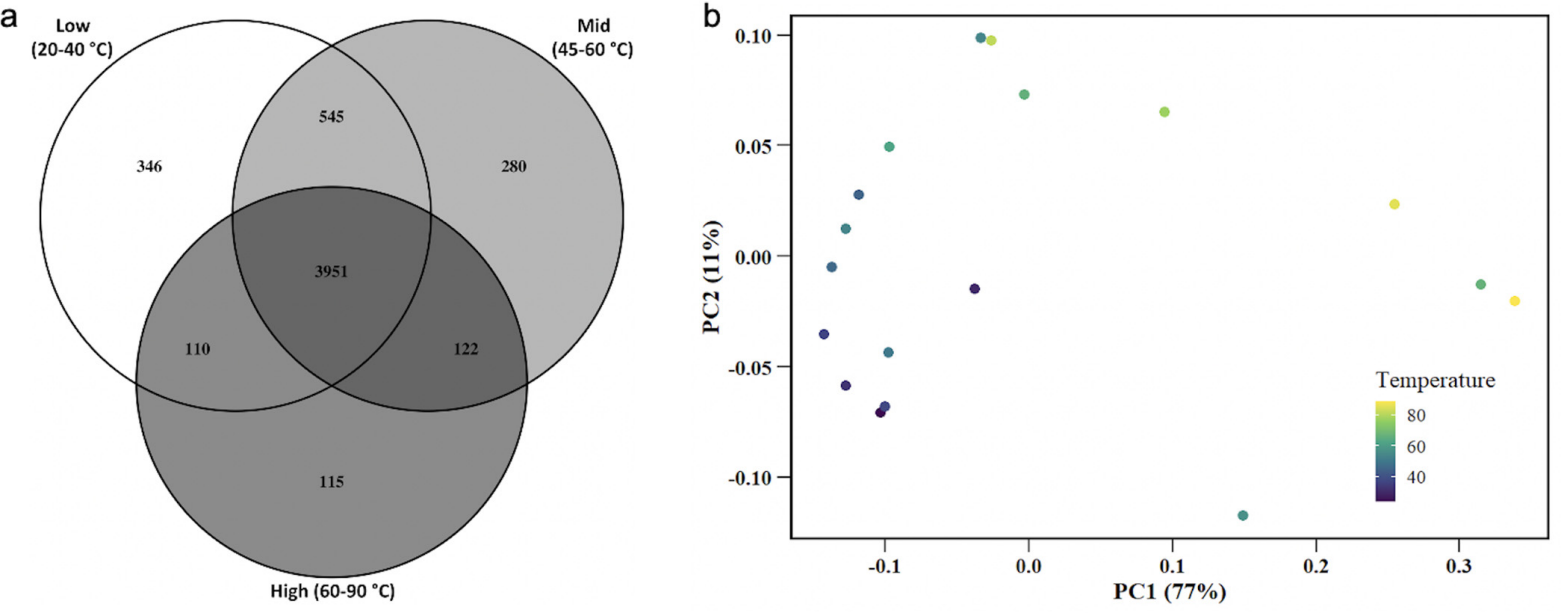
(a) Venn diagram comparing the number of Pfams detected in three pools of samples, grouped by temperature. Each pool included 6 samples each rarefied to 600,000 Pfam-assigned reads. Exact sample temperatures are listed in Table 3. (b) Principal-coordinate analysis (PCoA) plot showing samples separating by temperature, in degrees Celsius, along PC1.

The 18 metagenome samples were combined into 3 groups of low, moderate, and high temperature, with 6 samples per group. The average temperature (and range) of each pool was low, 30.5°C (21.2 to 37.9°C); moderate, 51.2°C (45.0 to 57.2°C); and high, 74.7°C (62.8 to 88.8°C). Of the 5469 Pfams detected across all samples, fewer were detected in the high-temperature group (4,288) than the moderate- or low-temperature groups (4,898 and 4,952, respectively). Most Pfams (3,951, or 72%) were present in all three temperature groups (Fig. 4a), but of the remaining temperature-dependent Pfams, many were found only in the low or the low- plus moderate-temperature groups (891), while fewer belonged to only the high- or the high- plus moderate-temperature groups (237). The low-temperature group contained the highest number of unique identified Pfams (346), followed by the moderate-temperature group (280) and then the high-temperature group (115). These trends all indicate that there was a greater pool of total Pfams at lower temperatures. Fewer total Pfams were found in the high-temperature group, and fewer Pfams were unique to this group than the others (Fig. 4a). Pfam beta diversity was also assessed with a principal-coordinate analysis (PCoA) plot. The PCoA showed samples broadly separating by temperature along principal coordinate 1 (PC1) (Fig. 4b), indicating that temperature has an effect on Pfam composition in these samples.

### Temperature dependence of individual Pfams.

The Pfam diversity indices in Fig. 3 reflect the combined trend of 5,469 Pfams detected across all samples. However, Pfams were also examined qualitatively to identify individual Pfams that showed clear upper temperature limits, i.e., that were abundant at lower temperatures but absent or very rare at higher temperatures (defined as 2 or fewer occurrences per 600,000 Pfam-assigned reads). Some of these are listed in Table S2. As expected from the overall Pfam diversity, which decreased most rapidly above 60°C (Fig. 2), many individual Pfams disappeared between 62 and 85°C. Notable trends included the disappearance of many phototrophy-associated Pfams at 62 to 67°C, but similar thresholds were also observed for Pfams representing other catabolic enzymes (e.g., multicopper oxidase, iron-only hydrogenase, and some glycosyl hydrolases), biosynthesis systems (e.g., gas vesicles, poly-β-hydroxybutyrate [PHB], and cobalamin), transporter elements, Cas elements, plasmid systems, and restriction enzymes (Table S2). Most of these Pfams are found in *Bacteria* and *Archaea*, or all three domains, but a few are Pfams exclusive to *Eukarya*. *Eukarya*-only Pfams generally disappeared abruptly at 62 to 66°C, in line with the presumed upper-temperature limit of eukaryotes at 62°C (31) (Table S2).

10.1128/mSystems.00991-21.8 TABLE S2 Selected Pfams that declined to 0 to 2 reads per 600,000 at 54 to 86°C. We assume that 1 to 2 Pfam reads could represent metagenomic reads obtained from contamination or dispersal of nonviable cells. The list is not intended to be exhaustive. The identities and distribution of Pfams across the three domains were determined with reference to the EMBL Pfam database (https://pfam.xfam.org). The temperature range in which the average number of reads declined to less than 2 reads per 600,000 is indicated by a black outline for each Pfam. The final two columns show the result of a Songbird analysis with samples divided into 2 temperature groups (<63°C and >66°C), which ranked the Pfams based on their temperature dependence. The selected Pfams are not necessarily expected to show the highest ranks of all Pfams, as the Songbird regression recodes 0s to a small positive number, and the temperature ceiling of all Pfams is not always the 63 to 66°C threshold used in the analysis. However, it does demonstrate that the selected Pfams in this table are usually in the top 10 to 20% most responsive Pfams. Download Table S2, PDF file, 0.1 MB. Copyright © 2022 Ruhl et al. 2022 Ruhl et al. https://creativecommons.org/licenses/by/4.0/ This content is distributed under the terms of the Creative Commons Attribution 4.0 International license.

Pfams predictive of sample temperature were also identified via random forest analysis, using either individual sample temperatures or samples grouped into three temperature ranges (Table S3). The Pfams identified as the strongest predictors generally declined at higher temperatures, although very few increased (e.g., PF00117, glutamine amidotransferase, and PF02554, carbon starvation protein CstA). Most Pfams declined continuously across the temperature gradient, while others showed temperature thresholds, reaching near 0 reads at some temperature above 55°C, similar to the Pfams identified in Table S2. Only a decline to 0 (as in Table S2) will cause a decrease in Pfam richness (observed_Pfam_ or Chao1_Pfam_) with temperature. However, the gradual decline in the abundance of many other Pfams with increasing temperature (Table S3) will also contribute to lower diversity and evenness indices at high temperatures (Shannon_Pfam_).

10.1128/mSystems.00991-21.9 TABLE S3 (Top) Fifty Pfams identified as important for temperature prediction in a random forest regression in order of importance. (Bottom) A second analysis using a random forest regression that removes singletons and groups Pfams into three temperature groups (highlighted blue, yellow, and orange) is also presented. A black outline indicates temperatures at which that Pfam declined to 0 to 2 reads per 600,000. Download Table S3, PDF file, 0.1 MB. Copyright © 2022 Ruhl et al. 2022 Ruhl et al. https://creativecommons.org/licenses/by/4.0/ This content is distributed under the terms of the Creative Commons Attribution 4.0 International license.

We note that the stresses in our sample set may not be limited to temperature. Multiple factors covary along with temperature in hot springs, notably oxidation state. However, aerobic capability across the entire temperature range was supported by the presence of multiple Pfams detected at all temperatures tested, including Bac_globin (PF01152), peroxidase (PF00141), protoglobin (PF11563), Bac_luciferase (PF00296), catalase (PF00199), homogentisate 1,2-dioxygenase HgmA (PF04209), and multiple components of cytochrome *c* oxidase (PF00115, PF00116, PF00510, PF03626, PF09125, and PF06481) (Table S2).

## DISCUSSION

In this study, we demonstrated that metagenomic functional diversity (FD) of microbial communities covaried with species diversity (SD) along a temperature gradient. As predicted, both measures declined as temperature increased. However, unlike linear FD versus SD trends observed previously in metagenomic data sets (21, 22, 24), we observed a concave-down power relationship. Pfam diversity increased rapidly when moving down the temperature gradient from hyperthermophilic to slightly cooler conditions (from 90 down to 60°C). This rapid increase in Pfam diversity appeared to be related to the appearance of many functions such as photosynthesis. However, below around 50°C, the relationship plateaued, and increasing species richness added very few new Pfams.

Emergent functional properties of communities are thought to depend on the diversity of organisms contributing to that function. A plot like Fig. 3, showing some index of functionality or functional diversity versus species diversity, is commonly called a biodiversity-ecosystem function (BEF) curve (32). A concave-downward power function like the one we observed is consistent with many experimental and theoretical models of BEF relationships in plant, animal, and microbial communities (20, 32–34). Often, such curves saturate quickly at low species richness (35). The logic of the concave-down relationship is that additions of taxa to simple communities add many new ecotypes or functions, but, as more species are added, functional saturation is approached, and further species additions primarily increase functional redundancy rather than functional capacity, a trend often termed the rivet hypothesis (33, 36). In our data set, this is consistent with most Pfams representing central metabolic and housekeeping functions that are common across species and therefore highly redundant (37–39). Only a few more specialized Pfam functions are introduced with increasing species richness.

This concave-down FD-SD power relationship in microbial communities has also been predicted in theoretical metagenome studies (29, 32, 33). Miki et al. (29) constructed orthologue accumulation curves from a set of microbial genomes and concluded that functional (orthologue) richness (MF) is a power function of species richness (SR), exactly as we observed in our data. From their model (MF = *c*SR*^a^*), *c* is the average number of functions in a single genome (1,190) (Fig. 3a), while the exponent *a* is a measure of community multifunctional redundancy, the degree to which functions are shared across genomes. A value of 0 would mean all genomes and communities are identical functionally, while a value of 1 would mean that each genome is totally unique and community functional diversity increases linearly with species diversity. The multifunctional redundancy in the field data (*a* = 0.164) was significantly less than in our artificial genome set (*a* = 0.077) (Fig. S3a in the supplemental material). However, this could be explained in part by the fact that thermophile genomes are smaller and contain fewer Pfams than mesophile genomes (see Fig. S4 for supporting data). In a power function, thermophiles will, therefore, display a lower factor *c* (average number of Pfams) than mesophiles. Combining a curve for high-diversity communities that are all mesophilic with a curve for low-diversity communities that are all thermophilic (as is the case in our field data) will flatten the apparent relationship and make it appear to have a higher exponent *a* than either curve alone. When we simulated this effect (Fig. S3b), the power fit coefficients for the simulated genome data set matched quite well to our experimental data. There are many sources of uncertainty in these calculations, primarily the incomplete sampling of rare Pfams via metagenomics. However, if Chao1 extrapolates reasonable estimates of community Pfams and species, these analyses suggest that the variability in Pfam diversity along the temperature gradient is directly related to species richness and genome size. Each new species added to a community introduces new Pfams in a predictable manner, so more species-rich communities are also predictably more function rich. The smaller size of thermophile genomes exaggerates the mathematical effect of the low species diversity in thermophile communities. A thermophile genome should contribute fewer new Pfams to a community than a mesophile genome simply because it has fewer Pfams, and the combination of fewer species with smaller genomes at high temperatures results in very low functional diversity.

While these considerations suggest that the lower FD is directly related to the lower community diversity and smaller genome sizes of thermophiles, they do not suggest causality, i.e., why are thermophiles less diverse and possess smaller genomes? There are many possible explanations for the low diversity of extremophile communities (see Sharp et al. [4] and Ruhl et al. [8] for reviews). One possibility is that extreme temperature imposes physical or evolutionary constraints on the presence of certain enzymes, which function only in limited temperature ranges. Therefore, the “pool” of functions available to thermophiles is smaller. This is consistent with our Venn analysis showing the fewest total Pfams in the high-temperature communities (Fig. 4). It is also evident in the analysis of individual Pfams across our temperature gradient, which revealed that many Pfams declined in abundance with increasing temperature (Tables S2 and S3). Several Pfams became rare or undetectable above temperatures between 62 and 85°C (Table S2). Previous research has noted that physical environmental factors can control the distribution of key functional clades within communities (25), and the existence of temperature ceilings for certain functions has been documented. For example, it has long been known that photosynthetic microbial mats disappear sharply above some temperatures between 60 and 73°C, depending on the geographic location of the spring (40). Indeed, the observed disappearance of many Pfams related to photosystems at high temperature (Table S2) was a conspicuous contributor to the overall FD trend in our data set, although this trend was not limited to photosynthesis-related Pfams (Table S2). Clearly, certain Pfams in our communities simply disappear at high temperatures, contributing to a low multifunctional redundancy across the entire data set.

While many experimental studies show concave-down BEF curves, field studies can occasionally show linear or concave-up curves instead (41), especially when measuring simple communities or specialized functions carried out by a few community members (29, 42, 43). The apparently linear FD versus SD curves observed in previous metagenome studies (21, 22, 24) could, therefore, be real. However, they are possibly also artifacts of a limited diversity range observed. Our sample set included SD values ranging over an order of magnitude from 400 to 4,000 OTUs, making the concave nature of the relationship evident. We also caution that there is a difference between measuring functional metagenome diversity and measuring emergent functional properties such as respiration. While collector's curves of gene orthologs or Pfams should be monotonic, emergent functional properties can be unpredictable. For example, more diverse communities may provide increased species connectivities, thereby altering BEF properties (41). Studies such as ours that quantify Pfams, KEGG pathways, or orthologues are reducing multidimensional niches (a combination of factors) to single dimensions (29, 39).

Generating our FD-SD temperature curves required a sample set covering a range of community diversities. For this, we chose samples varying in temperature since many studies have observed a relationship of microbial species diversity to temperature (4, 10–18). The Earth Microbiome Project has incorporated data from many environment types and inferred a general Gaussian relationship between temperature and alpha diversity (44). Several independent studies have observed a temperature-diversity trend in geothermal springs, either by comparing different springs (4) or comparing samples across temperature gradients within a single spring (10–12, 18). In contrast, a recent study of almost 1,000 geothermal springs in New Zealand reported that diversity was primarily correlated with pH and that temperature only had an effect above 70°C (5). However, when the effect of pH in this data set is removed (*r*^2^ = 0.19) (Fig. S5b), temperature does explain a significant portion of the residual variability across the entire temperature range of 20 to 100°C (*r*^2^ = 0.11) (Fig. S5d), and the temperature relationship is stronger when only neutral springs are considered (*r*^2^ = 0.29) (Fig. S5a). The weaker relationship between diversity and temperature observed by Power et al. (5) compared to other studies may result from inherently high geochemical diversity across sites in New Zealand, leading to a high residual variability unaccounted for by either pH or temperature (Fig. S5). In order to eliminate these complicating factors and ensure that diversity in our sample set was controlled by a single overriding factor, we selected samples from geochemically similar, pH-neutral springs. As a result, we observed extremely strong relationships (*r*^2^ between 0.71 and 0.90) between bacterial alpha diversity and temperature in both temperature transects from a single spring and across the entire subset of 9 different springs (Fig. 1). The relationships closely matched those reported by Sharp et al. (4) (Fig. S1), but with even higher *r*^2^ values of up to 0.90.

10.1128/mSystems.00991-21.5 FIG S5 OTU richness of *Bacteria* and *Archaea* versus temperature and pH in water samples analyzed by Power et al. (5). (a) OTU richness versus temperature of 470 water samples within a limited pH range (5 to 9). (b) OTU richness versus pH of the entire Power et al. (5) data set (925 samples). (c) Regression analyses of residuals of the plot in panel a versus pH. (d) Regression analyses of residuals of the plot in panel b plotted versus temperature. The solid lines represent the best-fit nonlinear exponential least-squares (a) or linear least-squares (b to d) regressions to the data. Data provided courtesy of the 1000 Springs Project team (5). Download FIG S5, PDF file, 0.9 MB. Copyright © 2022 Ruhl et al. 2022 Ruhl et al. https://creativecommons.org/licenses/by/4.0/ This content is distributed under the terms of the Creative Commons Attribution 4.0 International license.

Whether our observed relationship between SD and FD holds along diversity gradients related to other factors (e.g., pH, productivity, salinity, etc.) may depend on the underlying causes of these diversity gradients, which are not completely understood. Possible explanations include (i) a metabolic stress effect (as physical stress increases, more energy is needed for stress tolerance and repair, and, therefore, only increasingly high-energy metabolic lifestyles are possible), (ii) a physical stress effect (certain enzymes and metabolites become unstable and/or nonfunctional at high stresses), (iii) a niche availability effect (the number of species that have evolved to survive under certain conditions is dependent on the extent of that habitat geographically and historically), and (iv) a productivity effect (highly productive communities generally support more species) (45). Most of the above explanations would equally apply to species diversity gradients observed along environmental stresses other than temperature, such as pH (2–5), aridity (6), and salinity (7, 8). Indeed, recent studies suggest that functional diversity may also be reduced by salinity (46) and aridity (26).

While taxonomic diversity can be reliably estimated in microbial communities, there is no accepted universal measure of functional diversity, which requires a proxy index. Tools have been developed to predict community functionality based on correlating 16S rRNA genes to the known metabolic properties of cultured microbes (44, 47). However, these are dependent on the completeness of genome databases and give only indirect, and often probably incorrect, data. Given the high number of uncultured taxa in geothermal environments (48), there could also be systematic biases in applying these tools along a thermal gradient such as that in our study. Other studies have quantified gene orthologues from raw metagenome reads via BLAST. However, a BLAST approach, especially when using short sequence reads, is error prone and also likely prone to biases in database coverage of mesophilic versus thermophilic microbial communities. We therefore chose a Pfam-based approach, in part because these represent conserved functional or structural protein domain families (49), not specific proteins, and thus, a single Pfam may be present in psychrophilic, mesophilic, and thermophilic proteins (50). This suggests that the evolutionary adaptations involved in thermostability are not, on their own, sufficient to result in diversification of a new Pfam family. Therefore, quantitative surveys of Pfams are theoretically not sensitive to limitations imposed by fewer available reference sequences from extreme environments. In line with these predictions, we did not observe a decreasing relationship between the percentage of metagenome reads that were assigned a Pfam and sample temperature (Table 3). However, we cannot rule out that novel domains that are evolved only in thermal environments, with no mesophilic homologues, have been missed due to historical biases. Additionally, since individual Pfams represent protein domains, not complete metabolic pathways, a reduction in Pfam diversity with increasing temperature does not necessarily correlate with a reduction in the number of metabolic lifestyles in a community. Although Pfam diversity is only an index, we nevertheless feel the trends are illustrative.

In summary, this study documented three emergent patterns of alpha diversity. First, it verified previous studies showing that species diversity (SD) declines with increasing temperature in microbial communities. This temperature effect is very strong when complicating factors like pH are removed. Second, it demonstrated that community functional diversity (FD), measured as metagenomic Pfam diversity, also declines in response to increasing temperature stress. Third, it showed that the FD-SD relationship follows a concave-down power function, as has been predicted in metagenome models. The low FD in thermophilic communities is likely imposed by (i) a simple mathematical consequence of lower species diversity and smaller average genome sizes at high temperatures, which reduces the total metagenome size and therefore the number of (rare) Pfams; and (ii) physical, energetic, or evolutionary constraints that place upper-temperature limits on particular metabolic functions like photosynthesis. The first conclusion is supported because sampling of random genomes produces a similar power function in Pfam accumulation as seen in our field data. The second conclusion is supported by close examination of Pfam sets across temperatures, indicating that many Pfams do not occur in any high-temperature communities. These two factors may, in fact, be connected, i.e., compared to mesophiles, thermophile genomes may be smaller and thermophilic communities simpler because their pool of potential functions is smaller. However, other explanations for the trend are possible. Future studies quantifying metabolic capacity and redundancy along gradients of temperature and other stresses will be useful in elucidating these relationships.

## MATERIALS AND METHODS

### Sample collection.

Multiple samples were collected on 24 August 2015, from the Dewar Creek hot spring, located at 49°55′N, 116°28′W. At this site, pH-neutral meteoric water rises up to 5 km through silicate crustal rock and discharges at 83°C (51). The water cools gradually as it flows along outflow channels over a tufa mound (52). Twenty sediment samples were collected along the outflow channels, ranging in temperature from 24.2 to 79.8°C. Sediments were usually composed of sand-textured particles 1 mm or less in diameter, with occasional 5-mm or larger pebbles. Temperature was measured *in situ* and sediments removed to no more than 8 cm depth with a trowel, which was rinsed in stream water and ethanol between samples. Samples were stored at ambient temperature in 50-mL centrifuge tubes for less than 24 h during transport to the laboratory and then frozen at −20°C.

Additionally, 18 samples from 9 pH-neutral hot springs in Canada and New Zealand were selected from a previous study (4) (Table 1). The subset of springs was chosen to represent a wide temperature range of 21.2 to 88.8°C, but a narrow pH range (6.85 to 8.10). These samples had been collected between 2010 and 2012 and preserved at −80°C (with 5% dimethyl sulfoxide [DMSO] added). Temperature and pH measurements taken *in situ* are reported in Sharp et al. (4).

### 16S rRNA gene library preparation, sequencing, and analysis.

Samples were thawed at room temperature and homogenized in a Precellys 24 bead mill homogenizer (Bertin Instruments, Montigny-le-Bretonneux, France), and DNA extraction was performed using the FastDNA extraction kit for Soil (MP Biomedicals, Santa Ana, CA, USA) with the following modifications: an additional 5.5 M guanidine thiocyanate wash was performed (53), and elution was in Qiagen Elution Buffer (Qiagen, Toronto, Ontario, Canada). Extracted DNA was quantified using the Qubit HS kit (Invitrogen, Carlsbad, CA, USA) and diluted to 5 ng μL^−1^ prior to amplification using the universal *Bacteria*-specific 16S rRNA gene primers 341fw (5′-CCTACGGGNGGCWGCAG-3′) and 785rv (5′-GACTACHVGGGTATCTAATCC-3′) (54). Amplicons were prepared for sequencing on an Illumina MiSeq platform as described in Ruhl et al. (8).

Raw sequence data were demultiplexed, and the barcode sequences were removed and then analyzed using the Quantitative Insights Into Microbial Ecology (QIIME) pipeline versions 1.9.1 and 2020.11 (55). For QIIME 1.9.1 analyses, forward and reverse reads were paired using a minimum overlap of 20 bp. All paired reads with a Phred quality score below 20 were removed. Reads were clustered into OTUs at 97% similarity, and taxonomy of the OTUs was determined using the SILVA database, release 128 (56). The clustering of OTUs was preferred over single sequence variants because OTUs more closely approximate the bacterial species concept, where monophyletic groups with similar genomes are clustered into species. An OTU threshold of 97% was selected for consistency with a previous study (4), but the temperature-diversity relationship was consistent across multiple levels of OTU clustering and amplicon sequence variants (ASV) clustering (Fig. 1). The OTU tables were rarefied to 13,460 reads per sample, excluding three samples from the analysis, and core diversity metrics were calculated using QIIME (55). For QIIME 2020.11 analyses, sequences were imported into QIIME2, trimmed to excise adaptor sequences, quality assessed, and, subsequently, processed using an R package, DADA2, to denoise, join the paired reads, and remove the chimera (57). Taxonomic assignment of amplicon sequence variants (ASVs) or a feature table for all data sets was performed with the feature-classifier plugin (58) employing a naive Bayes classifier approach. The taxonomy classifier for the analysis was trained on the SILVA database, release 138 (56).

### Metagenomic library preparation and sequencing.

DNA samples were quantified in triplicate using the Qubit HS kit (Invitrogen, Carlsbad, CA, USA) and diluted to 0.2 ng μL^−1^ each. Metagenomic libraries were prepared as described in Illumina’s (Illumina Inc., San Diego, CA, USA) library preparation protocol (59), except samples were eluted in half the recommended volume of resuspension buffer to concentrate the libraries. Libraries were validated and average library size calculated using a Bioanalyzer (Agilent Technologies, Santa Clara, CA, USA) or 4200 TapeStation system (Agilent Technologies, Santa Clara, CA, USA) (Table 3). Library dilution and preparation for sequencing were performed as described previously (8). Libraries were sequenced using a MiSeq reagent kit v3, 600 cycles (Illumina; catalog number MS-102-3003) or a MiSeq reagent kit v2, 500 cycles (Illumina; catalog number MS-102-2003) (Table 3).

### Analysis of metagenomes for Pfam-based functional diversity.

One obstacle to quantifying genes across metagenomes is assembly bias. Full-length genes are easier to annotate than gene fragments, but assembling the metagenomes would lead to a systematic bias against identification of genes that do not assemble well for reasons such as low coverage or high strain microvariability (60). Full-length genes would be more prevalent in the simple communities, creating systematic biases in the percentage of identified genes per sample. Therefore, a raw-read approach was adopted here, using the longest-read Illumina platform available (MiSeq, 300 bp) in order to optimize Pfam assignments. Additionally, 300-bp MiSeq reads are likely to contain just a single open reading frame and to be associated with a single Pfam (61).

To make the metagenomic libraries searchable for Pfams, raw metagenomic reads were translated into amino acid sequences in all 6 reading frames using Transeq (62). Stop codon positions were translated as an “X” instead of the default “*” to facilitate downstream analyses. The translated sequences were then used as a query against version 31.0 of the Pfam database (28), using the hmmsearch program in the HMMER package (63), version 3.2.1. The Pfam hit with the lowest E value of the 6 frames was selected from the HMMER output data using R (64). Diversity analyses were run on data sets rarefied to 600,000 reads with assigned Pfams (referred to as “Pfam-assigned reads”).

In order to verify the Pfam assignments, a validation procedure was performed as described in Fig. S6 in the supplemental material. In brief, Pfam assignments were made to all sequences in the GenomeDatabase (https://sourceforge.net/projects/genomedatabase/), which contains amino acid sequences of one reference species from each cultured bacterial genus. Then, all Pfam-assigned reads from the metagenomes were searched via BLAST against the GenomeDatabase. When the top BLAST hit for a metagenome read was associated with the same Pfam as identified by HMMER directly on the raw metagenome read, that read was considered to be validated. The rationale for this validation was that the full-length reading frames of the GenomeDatabase are more reliably associated with Pfams by HMMsearch than are the 300-bp MiSeq reads. The validation procedure should also remove Pfams that are found only in *Eukarya* or *Archaea*. Although our species diversity estimates are based on *Bacteria-*specific 16S rRNA gene sequencing, the metagenomes may contain DNA from all three domains. Data were rarefied to 700,000 Pfam-assigned reads per sample, resulting in loss of one sample (Table S4).

10.1128/mSystems.00991-21.10 TABLE S4 The number of total forward (“F”) and reverse (“R”) reads obtained for each metagenome and the number and percentage of these reads remaining after the validation step. Download Table S4, PDF file, 0.03 MB. Copyright © 2022 Ruhl et al. 2022 Ruhl et al. https://creativecommons.org/licenses/by/4.0/ This content is distributed under the terms of the Creative Commons Attribution 4.0 International license.

### Analysis of metagenomic libraries for Pfams associated with signature genes.

RDP’s FunGene repository contains many enzymes important in major biogeochemical cycles (65). The associated protein products for each gene were searched against the Pfam database. Of the 93 signature genes provided by FunGene, 1 gene could not be associated with any Pfam, 2 genes had multiple associated Pfams, and 90 genes could be associated with a single Pfam. Only 56 of these 90 Pfams were unique, as some Pfams were associated with more than 1 signature gene. The data set of 600,000 Pfam-assigned reads per sample was screened for the 56 signature Pfams (Table S1) using R; core diversity metrics were calculated on the screened data sets with QIIME.

### Statistical analyses.

Regression analysis and curve fitting were performed using GraphPad Prism version 8.1.1 (GraphPad Software, La Jolla California USA). Residual plots of linear regressions usually showed a bias; therefore, a nonlinear Gaussian least-squares model (Y = a*exp(-(X-b)^2^/2c^2^)) was chosen for most plots, consistent with a previous study (4). Plots of Pfam diversity versus OTU diversity were fit using a power model (Y = aX^b^). PCoA of the Pfam data was performed using the vegan software package for R (version 2.5-7) (66) using the Bray-Curtis metric as the basis for calculation.

To perform the random forest analyses (Table S3), mathematical models were fit to the data using scikit-learn library in python (67). The Pfam data set was filtered for potential low-level sample contamination by subtracting 2 from the counts and setting Pfams with counts of 2 or less to 0. Continuous and discrete random forest models were fitted using RandomForestRegressor and RandomForestClassifier constructors, respectively. For the regressor model, the training/test samples were stratified into 5 equally spaced bins over 21 to 89°C. For the classifier model, sample stratification was performed according to the following temperature ranges: 21.2 to 37.9°C, 45.0 to 57.2°C, and 62.8 to 88.8°C. The optimal (nondefault) parameters were the following: n_estimators 200, random_state 42, min_samples_split 3, min_samples_leaf 1, max_features='sqrt', max_depth 12, bootstrap False. The variation (*r*^2^) explained by the random forest regressor on the test data was 0.76. For the classifier model, the three temperature categories’ *r*^2^ was 0.72. These *r*^2^ values were averages of 10 independent training/test data splits for each model. The average feature importances were then obtained from the optimal random forest models using the feature_importances command, and the highest values are reported in Table S3.

Pfams most responsive to temperature were also predicted in a Songbird analysis via differential ranking (DR) as described by Morton et al. (68) (Table S2). For this model, samples were grouped into 2 categories (<63°C, >66°C) to best determine Pfams that declined across the ca. 65°C temperature threshold that was suggested by the alpha diversity analysis. The min-feature-count was set to 10, decreasing the total number of Pfams in the analysis from 5,469 to 3,343.

### Data availability.

The raw reads and metadata for the 16S rRNA gene and metagenomic data sets generated for this study can be found in the SRA repository under accession number PRJNA779083. Pfam counts and the data analysis workflow for the random forest analysis are available at https://github.com/nyirock/random_forests_pfam.
