# Supplementary figures and images for: Microbial Functional Diversity Correlates with Species Diversity along a Temperature Gradient
Source: mSystems. 2022 Feb 15;7(1):e00991-21. doi: 10.1128/msystems.00991-21 (PMC8845567; doi:10.1128/msystems.00991-21)

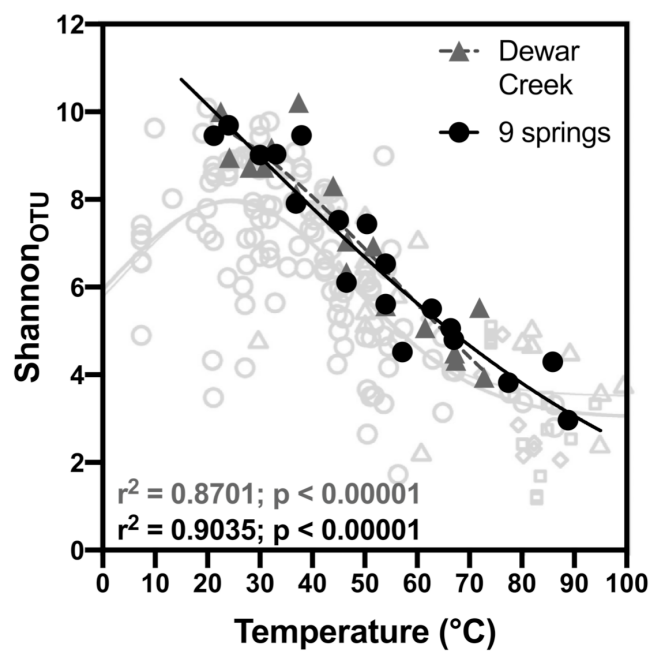

Supplement: FIG S1 [file msystems.00991-21-sf001.pdf]

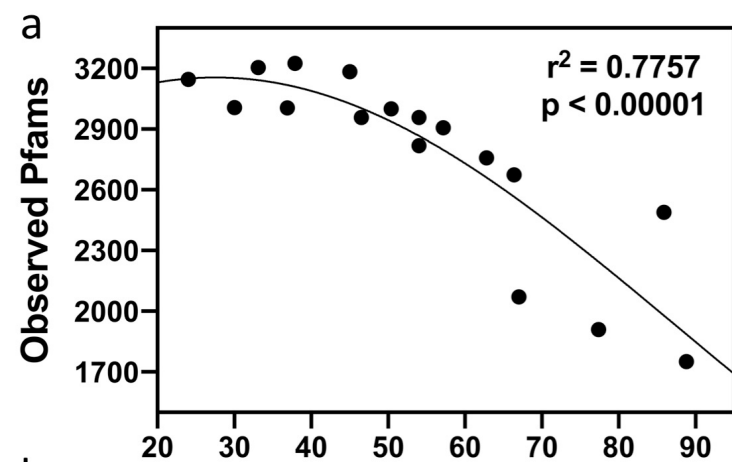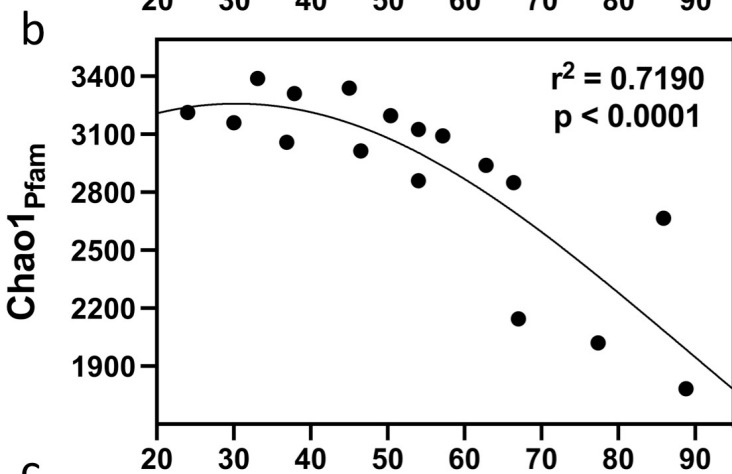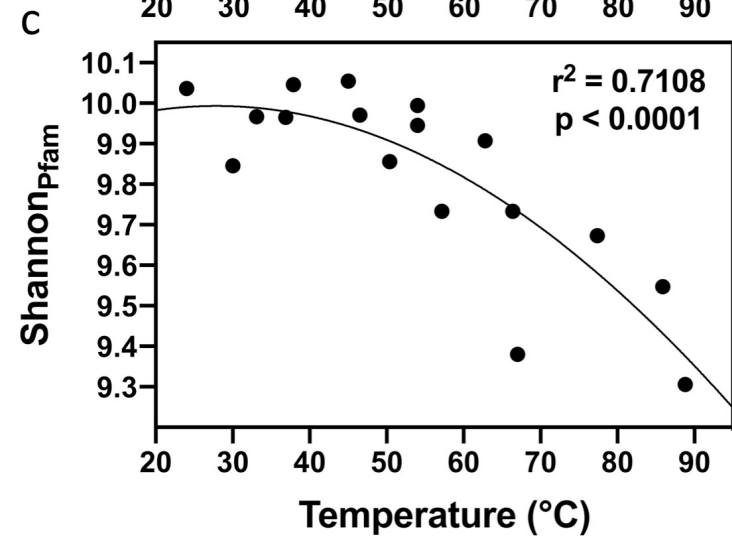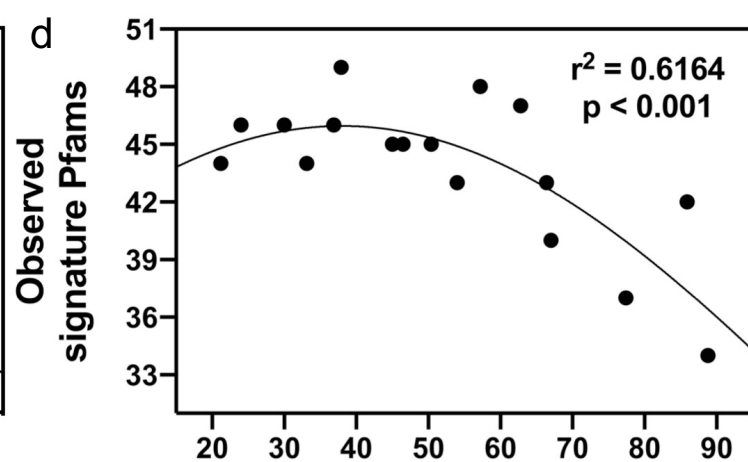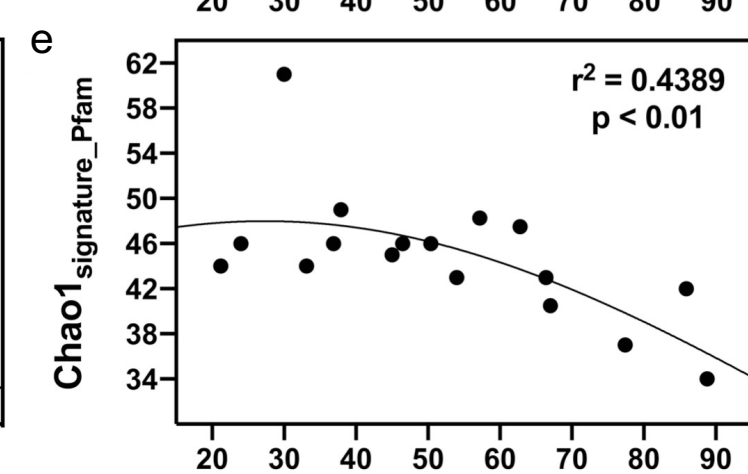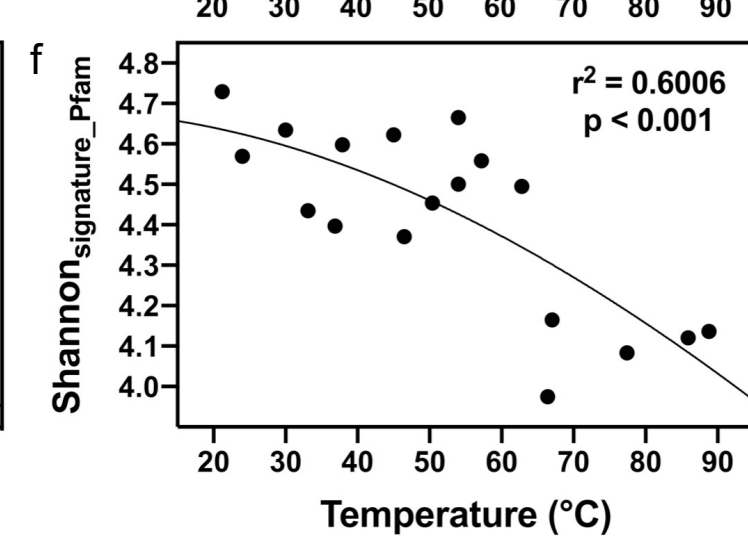

Supplement: FIG S2 [file msystems.00991-21-sf002.pdf]

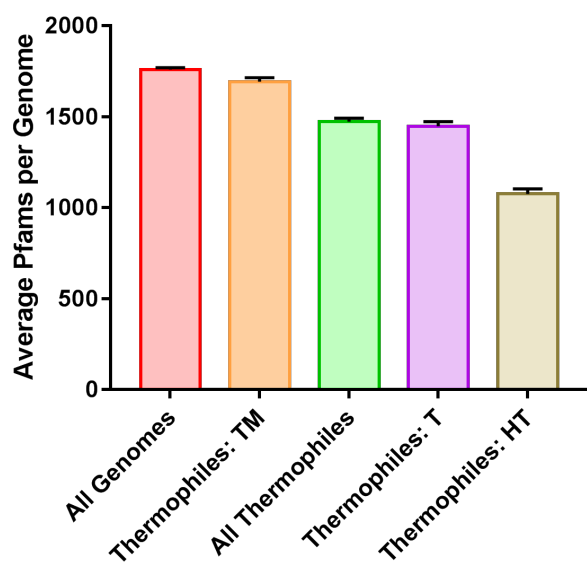

Supplement: FIG S4 [file msystems.00991-21-sf004.pdf]

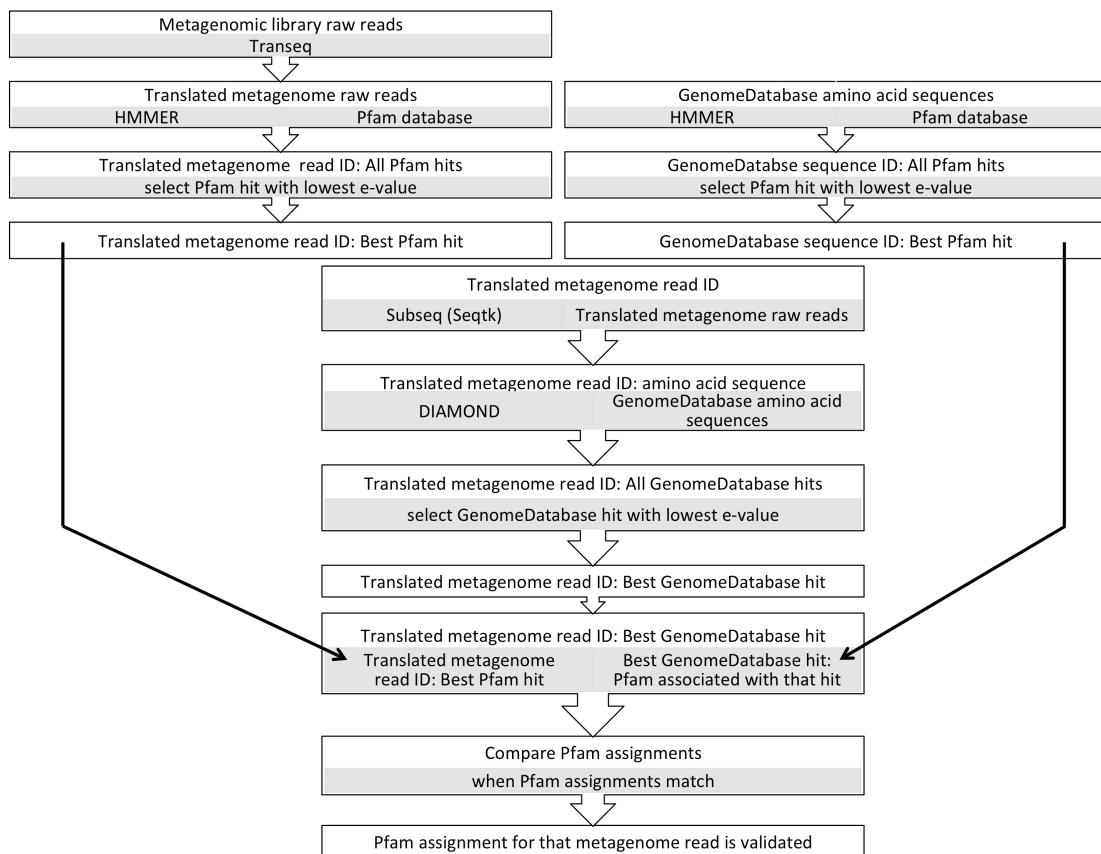

Supplement: FIG S6 [file msystems.00991-21-sf006.pdf]

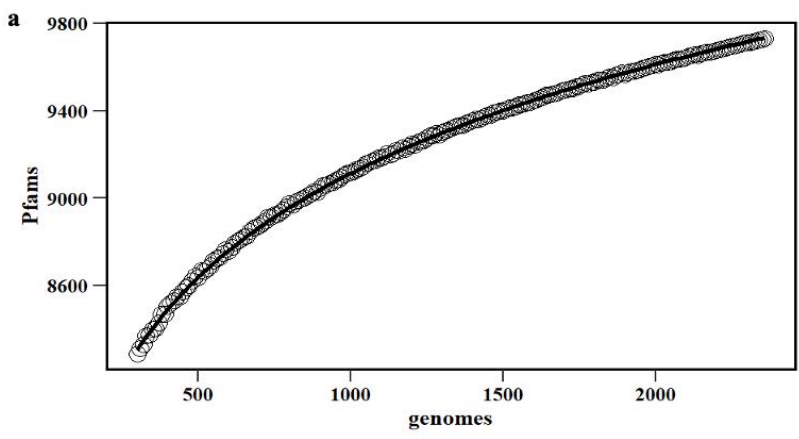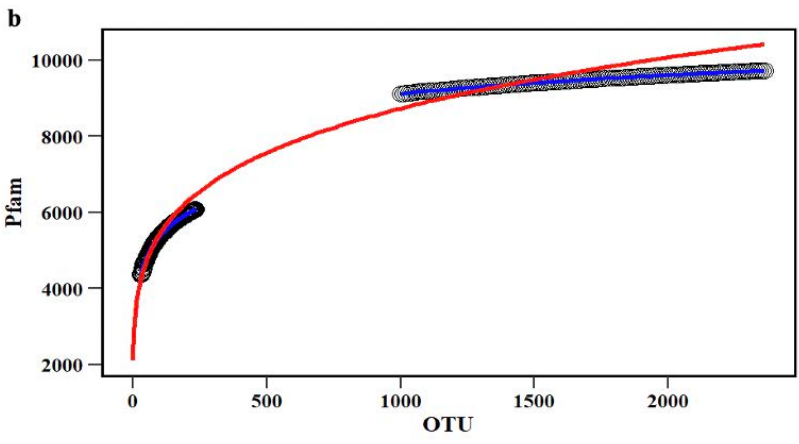

Supplement: FIG S3 [file msystems.00991-21-sf003.pdf]

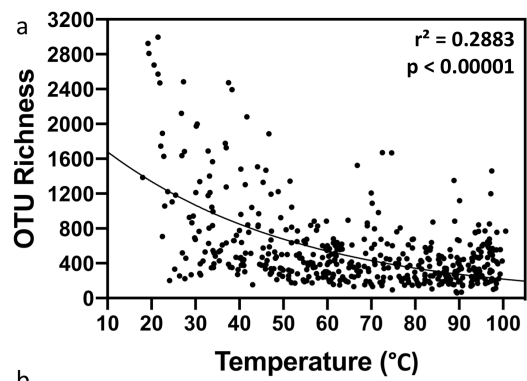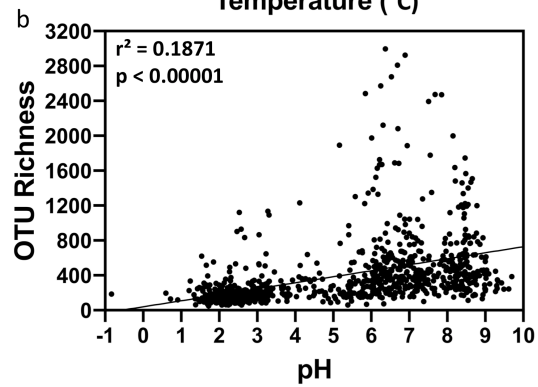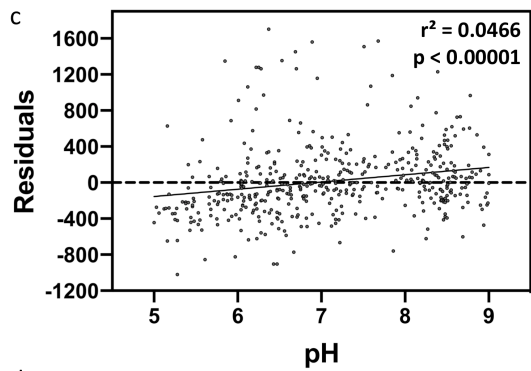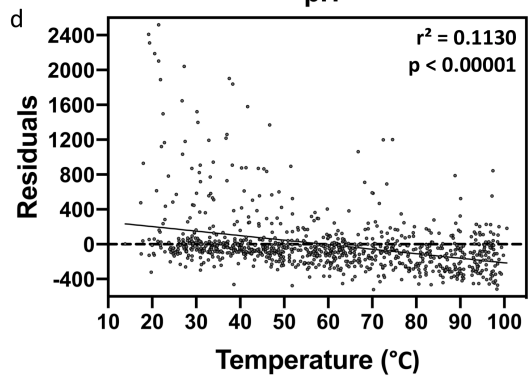

Supplement: FIG S5 [file msystems.00991-21-sf005.pdf]
