# Supplementary material for: Microbial Functional Diversity Correlates with Species Diversity along a Temperature Gradient
Source: mSystems. 2022 Feb 15;7(1):e00991-21. doi: 10.1128/msystems.00991-21 (PMC8845567; doi:10.1128/msystems.00991-21)
Supplement: TABLE S1 [file msystems.00991-21-st001.pdf]

Pfam ID	Pfam description	FunGene
PF00596	Aldolase_II	add1; add2
PF00245	Alk_phosphatase	ALP; alp_new
PF00795	CN_hydrolase	amiE
PF12942	Archaeal_AmoA	amoA_AOA
PF02461	AMO	amoA_AOB; amoA_AOB_like; amoA_comammox; NC10; pmo; pmoA_type1; pmoA_type1a; pmoA_type1c; pmoA_type2; pmoA_type2a; pmoA_type2b; pmoA_type3a; pmoA_type3b;
PF01112	Asparaginase_2/Asparagir	ansA
PF00206	Lyase_1	aspA
PF02333	Phytase	BPP
PF00871	Acetate_kinase	buk
PF02550	AcetylCoA_hydro	but
PF00840	Glyco_hydro_7	cbh1
PF03174	CHB_HEX_C	chb
PF08329	ChitinaseA_N	chiA
PF01752	Peptidase_M9	col
PF03063	Prismane	cooS
PF01546	Peptidase_M20	cpg
PF01654	Cyt_bd_oxida_I	cydA
PF01077	NIR_SIR	dsrA; dsrB; nirA; nirB
PF00728	Glyco_hydro_20	exc1
PF00115	COX1	fixN; norB
PF04960	Glutaminase	glsA
PF07250	Glyoxal_oxid_N	glx
PF00328	His_Phos_2	HAP
PF00221	Lyase_aromatic	hutH
PF02906	Fe_hyd_lg_C	hydA
PF07731	Cu-oxidase_2	lcc_ascomycetes; lcc_basidiomycetes; ppo
PF13417	GST_N_3	ligE
PF00141	peroxidase	lip; mnp; vp1
PF02249	MCR_alpha	mcrA
PF02332	Phenol_Hydrox	mmoX
PF00933	Glyco_hydro_3	nag3; nagZ
PF00384	Molybdopterin	napA
PF14710	Nitr_red_alph_N	narG
PF00148	Oxidored_nitro	nifD; vnfD
PF00142	Fer4_NifH	nifH
PF00394	Cu-oxidase	nir
PF13442	Cytochrome_CBB3	nirS
PF00116	COX2	nosZ; nosZ_atypical_1; nosZ_atypical_2
PF01447	Peptidase_M4	npr
PF02335	Cytochrom_C552	nrfA; nrfA_Welsh
PF13247	Fer4_11	nxrB; nxrB_nitrobacter; nxrB_nitrospira
PF00067	p450	p450nor
PF00883	Peptidase_M17	pepA
PF01433	Peptidase_M1	pepN
PF00171	Aldedh	phnX
PF09423	PhoD	phoD
PF02503	PP_kinase	PPK
PF00102	Y_phosphatase	PTP
PF00491	Arginase	rocF
PF00561	Abhydrolase_1	scd2
PF02872	5_nucleotid_C	soxB
PF00082	Peptidase_S8	sub
PF00089	Trypsin	trp
PF01979	Amidohydro_1	ureC
PF03139	AnfG_VnfG	vnfD_anfD
PF01261	AP_endonuc_2	xylA
