# Supplementary material for: Microbial Functional Diversity Correlates with Species Diversity along a Temperature Gradient
Source: mSystems. 2022 Feb 15;7(1):e00991-21. doi: 10.1128/msystems.00991-21 (PMC8845567; doi:10.1128/msystems.00991-21)
Supplement: TABLE S2 [file msystems.00991-21-st002.pdf]

Sample Temperature			21.2	24.0	30.0	33.1	36.9	37.9	45.0	46.5	50.4	54.0	54.0	57.2	62.8	66.4	67.0	77.4	85.9	88.8	Differential rank	Log-fold change
**1. Pfams found in all three domains *(Bacteria , Archaea , Eukarya)***																						
ACBP	Acyl CoA binding protein ACBP	(PF00887)	19	18	5	12	29	11	25	38	18	20	12	4	41	0	0	0	0	0	320	1.502
Amidoligase_2	Amidoligase	(PF12224)	27	2	13	15	9	21	7	1	8	4	6	16	1	0	0	0	0	0	755	0.978
Branch	Core-2/I-Branching enzyme Branch	(PF02485)	10	9	0	1	6	4	3	5	1	4	2	7	2	1	0	0	0	0	1264	0.472
Cauli_VI	Caulimovirus viroplasmin Cauli_VI	(PF01693)	14	8	2	10	17	4	21	36	12	33	17	5	37	4	1	0	0	0	495	1.264
Chal_sti_synt_C	Chalcone and stilbene synthases, C-terminal domain	(PF02797)	25	37	8	19	39	17	11	35	15	23	13	17	25	5	2	0	0	0	294	1.538
Chal_sti_synt_N	Chalcone and stilbene synthases, N-terminal domain	(PF00195)	14	11	8	6	35	22	8	16	5	10	12	7	26	9	1	0	1	0	610	1.124
CIA30	Complex I intermediate-associated protein 30	(PF08547)	42	16	33	18	16	5	29	16	6	17	7	87	7	0	0	0	0	0	497	1.262
CmcI	Cephalosporin hydroxylase	(PF04989)	25	52	31	36	38	75	28	24	70	21	24	5	7	7	0	0	1	0	226	1.652
COQ9	required for the biosynthesis of coenzyme Q	(PF08511)	19	11	2	3	7	4	7	11	2	10	1	7	0	0	0	0	0	0	466	0.708
CrtC	CrtC N-terminal lipocalin domain	(PF07143)	144	104	191	109	146	50	104	187	110	154	44	27	123	47	3	0	1	0	26	2.233
Cu-binding_MopE	Cu-binding_MopE	(PF11617)	78	82	123	56	76	65	45	18	38	26	10	20	3	5	0	0	0	0	234	1.637
Cu-oxidase	Multicopper oxidase	(PF00394)	15	11	3	22	20	43	12	27	28	24	25	31	5	2	2	1	0	0	435	1.342
Cyt-b5	Cytochrome b5-like Heme/Steroid binding domain	(PF00173)	12	30	16	6	5	17	18	12	5	4	11	6	14	0	0	0	0	0	557	1.194
DinB	damage-inducible (din) gene	(PF05163)	85	80	41	70	70	59	51	91	86	27	35	163	39	9	1	1	1	1	120	1.860
DNA_alkylation	DNA alkylation repair enzyme	(PF08713)	191	169	189	78	91	156	50	43	70	76	26	35	66	9	1	1	1	0	130	1.827
DUF3670	SNF2 Helicase protein	(PF12419)	16	20	22	30	23	29	14	17	6	38	7	81	10	6	5	0	0	0	582	1.161
Erythro_esteras	Erythromycin esterase	(PF05139)	59	184	23	157	40	124	23	32	62	20	19	26	2	7	0	0	0	0	161	1.770
Esterase_phd	Esterase PHB depolymerase	(PF10503)	32	41	9	18	25	50	19	4	23	11	5	13	7	8	0	0	0	0	291	1.540
ETC_C1_NDUFA4	ETC complex I subunit conserved region	(PF04800)	19	33	3	25	32	28	13	41	42	27	18	19	4	3	0	0	0	0	246	1.620
FA_desaturase_2	Fatty acid desaturase	(PF03405)	55	55	15	21	34	18	29	58	29	40	32	6	18	0	0	0	2	0	86	1.953
Fe_hyd_lg_C	Iron only hydrogenase large subunit, C-terminal domain	(PF02906)	242	142	128	97	14	256	200	27	83	50	125	3	78	4	8	0	0	2	137	1.813
Fe_hyd_SSU	Iron hydrogenase small subunit	(PF02256)	75	36	23	17	2	57	43	2	18	10	14	0	9	2	1	0	0	0	581	1.161
Glyco_hydro_32C	Glycosyl hydrolases family 32 C terminal	(PF08244)	16	6	5	1	6	12	10	19	5	19	5	1	25	11	0	0	0	0	863	0.872
Glyco_hydro_67M	Glycosyl hydrolase family 67 middle domain	(PF07488)	24	38	8	28	13	74	16	21	46	47	22	9	16	48	4	0	0	0	429	1.351
Glyco_hydro_8	Glycoside hydrolase family 8	(PF01270)	26	19	24	20	23	35	25	47	6	58	37	168	15	14	2	0	0	0	631	1.107
Glycolytic	Fructose-bisphosphate aldolase	(PF00274)	130	200	52	152	94	214	28	43	168	30	59	42	13	0	0	1	0	0	184	1.718
Heme_oxygenase	Heme oxygenase	(PF01126)	9	3	17	46	32	9	58	28	9	59	7	159	30	0	0	0	0	0	720	1.019
HYR	HYR domain	(PF02494)	61	33	24	68	97	43	29	151	14	171	26	54	22	30	1	0	1	0	181	1.723
ICMT	Isoprenylcysteine carboxyl methyltransferase (ICMT) family	(PF04140)	90	95	109	36	28	48	32	42	53	20	44	65	30	81	11	8	0	0	385	1.412
IDO	Indoleamine 2,3-dioxygenase	(PF01231)	18	15	5	21	26	17	6	7	15	39	19	20	17	7	0	0	0	0	271	1.584
KR	KR domain	(PF08659)	103	150	112	225	94	171	21	36	55	7	21	422	8	3	0	0	0	0	378	1.422
M16C_assoc	Peptidase M16C associated	(PF08367)	53	23	38	21	18	41	35	63	11	62	28	10	45	0	0	0	0	0	204	1.694
Malectin	Malectin domain	(PF11721)	61	76	73	15	86	27	26	103	43	146	21	34	196	3	0	0	0	0	243	1.624
MCD	Malonyl-CoA decarboxylase C-terminal domain	(PF05292)	19	41	16	56	49	44	18	17	127	13	61	13	3	0	0	0	0	0	313	1.517
PRiA4_ORF3	Plasmid pRiA4b ORF-3-like protein	(PF07929)	108	92	97	56	66	63	38	77	30	56	48	64	32	44	19	0	1	0	357	1.446
Pyridox_ox_2	Pyridoxamine 5'-phosphate oxidase	(PF12900)	97	94	117	78	39	61	47	10	53	6	19	127	20	9	2	0	0	2	177	1.730
SATase_N	Serine acetyltransferase, N-terminal	(PF06426)	30	31	1	28	9	40	6	16	43	20	13	22	1	1	0	0	1	0	339	1.475
Sod_Ni	Superoxide dismutase	(PF09055)	34	31	36	29	14	15	10	6	33	11	11	12	28	29	3	2	0	0	449	1.325
Sucrose_synth	Sucrose synthase	(PF00862)	75	32	16	96	32	8	73	39	0	11	7	77	1	3	0	0	0	0	295	1.537
SUFU	Suppressor of fused protein (SUFU)	(PF05076)	8	6	7	10	13	10	13	41	5	39	1	3	21	0	0	0	0	0	774	0.965
Sulfotransfer_1	Sulfotransferase domain	(PF00685)	37	37	21	20	13	33	13	12	16	13	7	55	5	2	0	0	0	0	387	1.412
TIG	IPT/TIG domain	(PF01833)	49	70	57	66	132	99	37	128	63	72	55	15	89	4	6	0	0	0	202	1.696
TLC	TLC ATP/ADP transporter	(PF03219)	32	77	11	9	6	79	1	1	4	3	7	20	0	0	0	0	0	0	682	1.051
TSP_3	Thrombospondin type 3 repeat	(PF02412)	44	25	18	8	37	14	20	7	2	26	12	15	14	7	0	0	0	0	356	1.446
Ubiq_cyt_C_chap	Ubiquinol-cytochrome C chaperone	(PF03981)	5	13	1	17	16	28	5	22	23	13	19	12	0	1	0	0	0	0	530	1.220
UDPGP	UTP--glucose-1-phosphate uridylyltransferase	(PF01704)	43	37	35	40	33	27	34	14	8	35	10	37	13	4	0	0	0	0	207	1.690
UnbV_ASPIC	ASPIC and UnbV	(PF07593)	136	157	82	123	171	119	66	95	104	99	53	159	97	58	3	0	1	0	22	2.288
VKG_Carbox	Vitamin K-dependent gamma-carboxylase	(PF05090)	58	68	3	54	45	108	7	24	43	15	3	39	11	13	0	0	0	0	203	1.695
VTC	VTC domain	(PF09359)	33	13	15	8	2	23	10	9	2	25	6	9	13	1	0	0	0	0	768	0.969
**1.2 Phototrophy proteins of *Bacteria* and *Eukarya***																						
Lycopene_cycl	Lycopene cyclase protein	(PF05834)	33	20	7	29	56	13	44	117	26	137	38	198	64	8	2	0	0	0	331	1.485
Mg-por_mtran_C	Magnesium-protoporphyrin IX methyltransferase C-terminus	(PF07109)	7	0	5	16	16	7	26	26	0	42	2	128	38	0	0	0	1	0	902	0.830
PCP_red	Proto-chlorophyllide reductase 57 kD subunit	(PF08369)	35	15	11	45	38	18	58	38	18	50	18	151	27	2	0	0	0	0	300	1.527
Photo_RC	Photosynthetic reaction centre protein	(PF00124)	112	28	58	202	305	58	307	390	41	486	32	1457	206	5	0	1	3	0	438	1.341
PHY	Phytochrome region PHY	(PF00360)	17	8	10	42	34	29	42	32	6	22	12	248	5	5	0	0	2	0	613	1.121
PRCH	Photosynthetic reaction centre, H-chain N-terminal region	(PF03967)	20	6	5	15	24	4	14	3	7	10	4	1	2	3	2	0	0	0	851	0.887
PsaA_PsaB	Photosystem I psaA/psaB protein	(PF00223)	4	1	48	188	214	32	221	164	16	394	15	1755	149	3	0	0	4	0	290	1.543
PsbH	Photosystem II 10 kDa phosphoprotein	(PF00737)	1	0	1	6	8	2	10	11	0	24	1	118	9	0	0	0	1	0	937	0.797
PsbM	Photosystem II reaction centre M protein	(PF05151)	1	0	2	4	8	0	26	11	0	28	0	56	13	0	0	0	0	0		
PsbU	Photosystem II 12 kDa extrinsic protein	(PF06514)	0	0	2	8	9	1	18	12	2	42	1	164	21	0	0	0	0	0	947	0.788
PSII	Photosystem II protein	(PF00421)	1	0	20	101	160	32	257	104	5	359	11	1366	130	4	0	0	2	0	661	1.079
**2. Pfams found almost exclusively in *Eukarya***																						
Actin	Actin	(PF00022)	3	42	4	8	14	38	4	2	12	6	4	21	33	0	0	0	0	0	331	1.485
Chitin_synth_2	Chitin synthase	(PF03142)	4	3	5	6	11	5	2	22	31	16	9	1	9	1	17	0	1	0	1981	-0.232
Chromo	CHRromatin Organisation MOdifier	(PF00385)	11	18	0	7	15	10	1	7	13	1	1	22	5	0	0	0	0	0	663	1.079
Cullin	Cullin	(PF00888)	7	8	0	0	3	7	0	2	6	4	2	9	18	0	0	0	0	0	1069	0.653
F-box-like	F-box-like	(PF12937)	9	26	0	8	1	24	1	20	60	14	3	12	5	0	0	0	0	0	751	0.653
I-set	Immunoglobulin I-set domain	(PF07679)	15	6	13	4	29	46	30	14	7	15	17	13	144	3	0	1	0	0	671	1.063
LIM	LIM domain	(PF00412)	4	5	0	5	1	8	1	9	11	1	1	7	16	0	0	0	0	0	1119	0.614
Mito_carr	Mitochondrial carrier protein	(PF00153)	11	20	3	9	13	33	3	1	43	3	1	28	16	0	0	0	0	1	555	1.194
**3. Pfams found alsmost exclusively in *Bacteria* and *Archaea***																						
Alph_Pro_TM	Putative transmembrane protein Alph_Pro_TM (PF09608)	(PF09608)	47	29	10	53	37	40	28	37	82	38	33	23	3	4	2	0	0	0	272	1.579
AmoC	ammonia/methane monooxygenase subunit C	(PF04896)	3	19	3	3	8	9	11	6	2	0	46	1	0	1	1	0	0	0	953	0.786
ChaB	cation transporter ChaB	(PF06150)	27	20	9	28	19	30	2	5	13	2	3	73	0	3	0	1	0	0	664	1.079
Chrome_Resist	Chromate resistance exported protein (PF09828)	(PF09828)	33	70	96	84	31	72	20	12	84	2	25	3	2	23	0	0	0	0	293	1.538
CitF	Citrate lyase, alpha subunit	(PF04223)	92	70	30	15	2	50	58	2	4	18	39	2	37	4	0	0	0	0	349	1.455
CobS_N	Cobaltochelatase CobS subunit N terminal	(PF12556)	22	18	8	14	15	18	7	13	31	5	11	6	2	0	0	0	0	0	538	1.212
CobT	Cobalamin biosynthesis protein CobT	(PF06213)	24	23	3	39	24	36	16	21	53	16	14	11	1	2	0	0	0	0	333	1.481
CobT_C	Cobalamin biosynthesis protein CobT VWA domain	(PF11775)	72	63	5	48	81	54	23	41	74	43	16	28	6	4	0	0	0	0	95	1.928
DCD	2'-deoxycytidine 5'-triphosphate deaminase	(PF06559)	80	113	9	100	87	59	20	91	95	84	55	37	2	7	2	0	0	0	98	1.906
DDE_Tnp_ISAZ013	Rhodopirellula transposase DDE domain	(PF07592)	497	482	513	166	454	162	8	387	100	166	16	41	3	3	0	0	0	0	362	1.441
DegS	Sensor protein DegS	(PF05384)	12	8	1	10	1	7	1	7	4	6	6	1	4	8	4	0	0	0	1566	0.170
Dehydratase_LU	trimeric diol dehydratases and glycerol dehydratase large subunit	(PF02286)	44	61	41	34	85	49	7	131	23	71	20	8	41	1	0	0	0	0	201	1.696
Dehydratase_MU	trimeric diol dehydratases and glycerol dehydratase medium subunit	(PF02287)	8	22	5	11	25	7	2	26	6	10	20	5	1	7	0	0	0	0	687	1.047
Dehydratase_SU	trimeric diol dehydratases and glycerol dehydratase small subunit	(PF02288)	12	14	4	8	7	13	0	15	2	8	1	0	7	2	1	0	0	0	1183	0.545
DHC	Dihaem cytochrome c	(PF09626)	13	9	31	10	7	5	25	8	7	19	4	44	12	0	0	0	0	0	603	1.135
DUF3731	DNA-K related protein	(PF12531)	18	24	20	21	31	25	22	24	10	19	20	6	12	13	0	0	0	0	274	1.575
EutA	Ethanolamine utilisation protein EutA	(PF06277)	37	67	34	131	24	44	18	6	196	7	70	2	14	65	6	2	0	0	500	1.256
Fib_succ_major	Fibrobacter succinogenes major domain	(PF09603)	202	126	103	3	7	44	9	1	0	0	0	9	7	10	0	0	0	0	770	0.967
FYDLN_acid	Protein of unknown function	(PF09538)	20	22	4	16	21	14	15	23	16	18	13	10	1	1	0	0	0	0	405	1.379
Gas_vesicle	Gas vesicle protein	(PF00741)	6	6	12	41	12	18	30	26	10	68	2	158	21	6	0	0	0	0	879	0.852
GldM_C	GldM C-terminal domain	(PF12080)	21	21	1	5	17	8	14	24	8	15	5	7	17	0	0	0	0	0	448	1.326
GldM_N	GldM N-terminal domain	(PF12081)	12	19	0	5	13	12	7	7	12	15	1	7	14	1	0	0	0	0	619	1.117
Gmad2	Immunoglobulin-like domain of bacterial spore germination	(PF10648)	13	18	14	16	7	26	9	16	21	21	5	3	5	7	0	0	0	0	660	1.082
GSCFA	GSCFA family	(PF08885)	44	53	9	27	34	33	11	40	11	49	12	16	57	7	0	0	1	0	241	1.627
GvpL_GvpF	Gas vesicle synthesis protein GvpL/GvpF	(PF06386)	8	12	8	72	15	36	55	39	10	45	9	316	29	4	2	0	0	0	406	1.376
HWE_HK	HWE histidine kinase	(PF07536)	53	73	6	77	29	113	32	62	69	71	22	8	0	9	0	0	0	0	215	1.678

ImpE	ImpE protein	(PF07024)	13	7	1	11	18	7	3	7	6	0	3	1	2	0	0	0	0	0	930	0.802
Intg_mem_TP0381	Integral membrane protein	(PF09529)	38	37	30	20	20	27	43	12	26	44	18	8	72	5	0	1	1	0	186	1.716
LAB_N	Lipid A Biosynthesis N-terminal domain	(PF07578)	34	35	31	29	49	23	19	30	16	13	18	5	47	3	0	0	0	0	235	1.635
LACT	Lecithin:cholesterol acyltransferase	(PF02450)	18	15	16	1	3	15	13	20	8	31	4	4	21	1	0	0	0	0	710	1.028
Methyltransf_14	C-methyltransferase C-terminal domain	(PF08484)	108	164	107	122	80	95	57	62	105	42	79	29	15	6	1	0	1	0	71	2.004
MltA	MltA specific insert domain	(PF03562)	43	54	53	70	67	43	54	34	61	33	38	88	10	10	0	0	4	0	36	2.164
NHase_alpha	Nitrile hydratase, alpha chain	(PF02979)	32	64	9	94	71	48	19	29	134	15	41	379	8	15	0	0	0	0	359	1.445
NHase_beta	Nitrile hydratase beta subunit	(PF02211)	20	51	1	83	39	43	18	18	109	19	18	418	3	22	2	0	0	0	693	1.045
PAS_5	PAS domain	(PF07310)	9	18	1	25	7	21	3	3	19	8	4	12	0	0	0	0	0	0	712	1.026
PHB_acc	PHB accumulation regulatory domain	(PF05233)	27	24	3	21	33	23	23	32	50	16	12	7	2	0	0	0	0	0	380	1.417
PHB_acc_N	PHB/PHA accumulation regulator DNA-binding domain	(PF07879)	71	68	43	55	70	52	48	81	76	77	40	29	43	8	1	0	0	0	28	2.230
PHB_depo_C	PHB de-polymerase C-terminus	(PF06850)	96	104	13	70	98	81	59	77	135	58	49	20	7	6	1	0	0	0	91	1.944
PHBC_N	Poly-beta-hydroxybutyrate polymerase N terminal	(PF12551)	12	9	2	10	2	7	17	5	15	3	10	1	3	0	1	0	0	0	838	0.906
Pilus_CpaD	Pilus biogenesis CpaD protein	(PF09476)	32	22	2	42	19	29	14	25	39	15	14	10	3	1	0	0	0	0	353	1.450
Porin_2	Porin subfamily	(PF02530)	28	13	1	28	2	23	11	18	48	7	21	2	0	0	0	0	0	0	536	1.214
Recombinase	Recombinase	(PF07508)	199	133	141	143	76	163	60	54	114	30	67	71	25	9	28	0	2	2	350	1.455
RhaT	L-rhamnose-proton symport protein (RhaT)	(PF06379)	25	25	26	9	12	22	9	3	9	3	1	0	2	1	0	0	1	0	771	0.966
Sulphotransf	Stf0 sulphotransferase	(PF09037)	10	18	11	9	11	15	6	20	9	26	0	11	17	0	0	0	0	0	615	1.119
Unstab_antitox	Putative addiction module component	(PF09720)	120	102	97	37	68	56	28	32	48	26	9	19	17	0	0	0	2	0	173	1.740
Usg	Usg-like family	(PF06233)	45	25	4	25	29	44	19	18	40	11	13	16	3	1	0	0	1	0	232	1.638
UxuA	D-mannonate dehydratase (UxuA)	(PF03786)	154	127	77	77	152	109	50	198	119	153	29	83	52	80	1	1	1	0	68	2.012
YTV	YTV	(PF07639)	47	123	22	35	60	108	7	92	20	16	15	17	6	17	5	0	0	0	576	1.168
**3.2 Bacterial phototrophy proteins**																						
KaiB	Cyanobacterial clock protein	(PF07689)	33	50	60	42	29	58	41	29	9	71	9	256	33	0	0	0	0	0	591	1.150
LHC	Antenna complex alpha/beta subunit	(PF00556)	34	15	4	5	22	10	23	23	5	29	5	13	24	1	0	0	0	0	430	1.350
PBS_linker_poly	Phycobilisome Linker polypeptide	(PF00427)	3	1	30	186	118	6	249	100	8	197	9	1064	78	3	0	0	2	0	501	1.256
**3.3 Restriction enzymes**																						
BsuBI_PstI_RE	BsuBI/PstI	(PF06616)	36	42	51	26	54	17	73	112	15	138	4	27	99	6	0	0	0	0	263	1.595
RE_CfrBI	CfrBI restriction endonuclease	(PF09516)	9	8	13	3	4	4	28	21	31	45	8	0	186	33	0	0	0	0	885	0.847
RE_Eco29kI	Eco29kI restriction endonuclease	(PF09517)	7	11	6	9	12	7	5	38	12	44	4	2	21	0	0	0	1	0	685	1.049
RE_XamI	XamI restriction endonuclease	(PF09572)	13	18	15	18	25	8	44	4	20	15	20	9	9	0	2	0	0	0	414	1.364
XhoI	Restriction endonuclease XhoI	(PF04555)	27	20	40	18	32	10	25	28	62	9	16	6	7	4	0	0	0	0	369	1.430
**3.4 Plasmid-related proteins**																						
Plasmid_killer	RelE-like toxin of type II toxin-antitoxin system HigB	(PF05015)	168	210	195	87	59	72	46	20	124	14	54	97	6	3	0	0	1	0	250	1.611
Relaxase	Relaxase/Mobilisation nuclease domain	(PF03432)	23	17	7	12	7	15	9	2	16	6	5	9	0	1	0	2	0	0	756	0.978
Rep_trans	Replication initiation factor	(PF02486)	11	3	0	2	1	4	77	18	3	25	3	4	27	8	0	1	0	0	970	0.770
RepA_C	Plasmid encoded RepA protein	(PF04796)	10	7	3	5	18	22	9	20	3	14	8	5	5	1	0	0	0	0	823	0.920
RepB	RepB plasmid partitioning protein (PF07506)	(PF07506)	28	10	6	10	10	8	5	4	3	0	1	2	0	0	0	0	0	0	1010	0.717
**3.5 CRISPR-Cas elements**																						
Cas_Cas2CT1978	CRISPR-associated protein (Cas_Cas2CT1978)	(PF09707)	10	18	15	9	9	10	12	9	13	32	21	2	25	8	3	4	0	0	976	0.759
Cas_Csd1	CRISPR-associated protein (Cas_Csd1)	(PF09709)	75	79	94	98	64	52	120	134	119	163	94	21	355	28	2	1	2	0	138	1.813
Cas_CT1975	CT1975-like protein	(PF09344)	24	37	45	32	29	31	50	21	50	95	68	16	34	36	9	35	0	0	1309	0.426
Cas_GSU0054	CRISPR-associated protein, GSU0054 family	(PF09609)	4	15	6	32	83	16	15	90	69	38	24	9	47	25	0	0	0	0	233	1.637
																					497<br>Avg.	1.31<br>Avg.
