# Supplementary material for: Microbial Functional Diversity Correlates with Species Diversity along a Temperature Gradient
Source: mSystems. 2022 Feb 15;7(1):e00991-21. doi: 10.1128/msystems.00991-21 (PMC8845567; doi:10.1128/msystems.00991-21)
Supplement: TABLE S3 [file msystems.00991-21-st003.pdf]

1. Random forest perfomred on individual metagenome samples

Sample Temperature			21.2	24.0	30.0	33.1	36.9	37.9	45.0	46.5	50.4	54.0	54.0	57.2	62.8	66.4	67.0	77.4	85.9	88.8
HlyIII	Haemolysin-III related	(PF03006)	126	146	70	91	61	154	96	47	75	40	40	30	41	16	4	8	13	0
RepB	RepB plasmid partitioning protein	(PF07506)	28	10	6	10	10	8	5	4	3	0	1	2	0	0	0	0	0	0
NHL	NHL repeat	(PF01436)	274	294	427	295	303	604	404	320	269	305	175	95	247	255	44	133	62	4
Unstab_antitox	Putative addiction module component	(PF09720)	120	102	97	37	68	56	28	32	48	26	9	19	17	0	0	0	2	0
Asp-Al_Ex	Predicted Permease Membrane Region	(PF06826)	54	82	92	31	54	99	55	26	57	42	36	12	46	7	0	1	7	0
Cu-binding_MopE	Putative metal-binding motif	(PF11617)	78	82	123	56	76	65	45	18	38	26	10	20	3	5	0	0	0	0
FtsX	FtsX-like permease family	(PF02687)	1493	1307	1297	1195	1217	1330	891	946	1067	792	817	726	967	674	718	634	505	578
HA2	Helicase associated domain (HA2)	(PF04408)	28	24	16	24	33	32	28	30	32	11	18	17	12	7	0	5	9	0
CotH	CotH kinase protein	(PF08757)	287	201	135	123	126	161	94	91	58	101	71	42	73	12	2	0	1	0
PBP5_C	Penicillin-binding protein 5, C-terminal domain	(PF07943)	19	15	21	27	30	41	22	19	22	3	17	7	8	3	4	0	10	0
CoA_trans	Coenzyme A transferase	(PF01144)	228	233	287	315	338	293	291	277	400	184	171	75	187	200	77	103	159	110
LZ_Tnp_IS66	Transposase C of IS166 homeodomain	(PF13007)	66	54	23	24	19	40	26	29	41	20	8	17	2	4	0	0	15	0
PA14	PA14 domain	(PF07691)	119	104	151	115	158	139	87	157	82	166	86	74	149	55	16	45	27	16
PTR2	POT family	(PF00854)	86	75	21	67	78	71	76	56	64	77	40	39	15	13	6	0	12	0
Phage_T4_gp19	T4-like virus tail tube protein gp19	(PF06841)	103	117	66	130	73	108	27	121	73	113	24	37	72	22	1	1	5	0
Porin_O_P	Phosphate-selective porin O and P	(PF07396)	51	51	33	40	46	76	43	43	37	30	16	18	28	19	0	3	18	0
EHN	Epoxide hydrolase N terminus	(PF06441)	25	61	14	62	30	80	7	7	55	2	12	6	1	1	0	0	0	0
AmoC	Ammonia monooxygenase/methane monooxygenase, subunit C	(PF04896)	3	19	3	3	8	9	11	6	2	0	46	1	0	1	1	0	0	0
PDEase_II	cAMP phosphodiesterases class-II	(PF02112)	9	10	10	4	2	13	5	4	6	5	5	1	2	0	0	0	0	0
ALO	D-arabinono-1,4-lactone oxidase	(PF04030)	34	36	5	28	16	43	10	6	44	21	8	20	10	4	0	0	1	0
SUFU	Suppressor of fused protein (SUFU)	(PF05076)	8	6	7	10	13	10	13	41	5	39	1	3	21	0	0	0	0	0
PMI_typeI	Phosphomannose isomerase type I	(PF01238)	49	64	24	15	16	33	11	19	27	18	9	9	22	1	0	1	7	0
Arabinose_Iso_C	L-arabinose isomerase C-terminal domain	(PF11762)	49	30	15	16	9	30	9	9	10	8	7	1	2	4	0	0	1	0
TctB	Tripartite tricarboxylate transporter TctB family	(PF07331)	56	63	343	136	64	88	84	35	223	34	79	12	31	47	11	1	7	1
Resolvase	Resolvase, N terminal domain	(PF00239)	577	534	363	419	246	576	214	199	278	118	175	159	101	43	104	64	175	780
Stig1	Stigma-specific protein, Stig1	(PF04885)	7	7	6	12	9	24	5	13	7	8	3	4	0	0	0	0	0	0
AIPR	AIPR protein	(PF10592)	103	104	34	42	47	56	57	24	53	3	13	31	8	2	4	0	2	0
Polysacc_deac_1	Polysaccharide deacetylase	(PF01522)	392	440	453	415	367	447	302	416	452	338	290	255	271	312	122	263	249	168
Dioxygenase_C	dioxygenase	(PF00775)	101	153	31	204	129	192	74	116	205	71	82	45	30	25	1	0	6	0
Autotrns_rpt	Passenger-associated-transport-repeat	(PF12951)	73	115	66	58	103	169	49	29	42	45	59	25	29	22	2	0	0	0
CHMI	5-carboxymethyl-2-hydroxymuconate isomerase	(PF02962)	9	3	14	5	2	4	1	4	8	1	1	1	0	0	0	0	0	0
PPK2	Polyphosphate kinase 2 (PPK2)	(PF03976)	653	716	406	357	398	471	351	301	362	244	185	304	232	27	0	0	43	1
PAF-AH_p_II	Platelet-activating factor acetylhydrolase, isoform II	(PF03403)	10	6	7	4	10	12	8	7	4	7	2	35	4	1	0	0	0	0
MlrC_C	MlrC C-terminus	(PF07171)	45	38	60	43	94	38	27	108	65	62	14	32	13	13	8	2	0	2
ABC_transp_aux	ABC-type uncharacterized transport system	(PF09822)	212	147	198	126	252	141	195	225	176	188	148	152	256	51	17	72	64	13
PT	PT repeat	(PF04886)	13	12	4	3	54	6	54	1	11	2	2	12	1	0	0	0	0	0
Methyltransf_14	C-methyltransferase C-terminal domain	(PF08484)	108	164	107	122	80	95	57	62	105	42	79	29	15	6	1	0	1	0
NDUFA12	NADH dehydrogenase (ubiquinone)	(PF05071)	16	13	3	36	26	19	12	18	23	14	4	4	1	1	0	0	0	0
BMFP	Membrane fusogenic activity	(PF04380)	38	31	3	25	18	36	25	37	42	19	22	7	4	6	0	0	16	0
ORF6N	ORF6N domain	(PF10543)	106	82	147	42	14	25	11	21	50	15	11	7	0	0	0	0	0	0
OST_LOTUS	OST-HTH/LOTUS domain	(PF12872)	31	27	14	12	5	13	12	4	12	4	13	6	0	1	0	0	1	0
RhaT	L-rhamnose-proton symport protein (RhaT)	(PF06379)	25	25	26	9	12	22	9	3	9	3	1	0	2	1	0	0	1	0
zf-NADH-PPase	NADH pyrophosphatase zinc ribbon domain	(PF09297)	8	14	7	11	18	14	7	18	10	14	6	5	6	0	0	0	3	0
ATPase-cat_bd	Putative metal-binding domain of cation transport ATPase	(PF12156)	25	17	7	19	26	7	25	7	22	7	11	7	10	3	0	0	4	0
Arylsulfotrans	Aryl sulfotransferase	(PF05935)	35	27	13	10	17	27	20	11	12	5	0	5	7	1	0	0	2	0
Glutaredoxin	Glutaredoxin	(PF00462)	163	192	134	128	142	160	185	120	129	150	162	373	120	48	85	76	48	6
iPGM_N	BPG-independent PGAM N-terminus (iPGM_N)	(PF06415)	201	265	97	197	176	202	234	175	119	181	102	256	164	64	9	90	26	0
2-ph_phosp	2-phosphosulpholactate phosphatase	(PF04029)	112	86	117	110	127	113	118	106	91	135	109	240	129	66	18	102	53	64
NMO	Nitronate monooxygenase	(PF03060)	235	245	280	260	199	258	209	134	310	154	190	74	153	112	60	187	88	73
ACC_central	Acetyl-CoA carboxylase, central region	(PF08326)	2	1	1	1	1	3	1	1	1	0	0	0	0	0	0	0	0	0

2. Random forest perfomred on metagenome samples grouped into three temperature groups

Sample Temperature			21.2	24.0	30.0	33.1	36.9	37.9	45.0	46.5	50.4	54.0	54.0	57.2	62.8	66.4	67.0	77.4	85.9	88.8
PRA-CH	Phosphoribosyl-AMP cyclohydrolase	(PF01502)	136	150	110	156	161	106	188	197	166	239	176	162	192	268	170	217	317	231
CotH	CotH kinase protein	(PF08757)	287	201	135	123	126	161	94	91	58	101	71	42	73	12	2	0	1	0
CutC	CutC family	(PF03932)	34	32	10	30	50	38	16	9	22	25	8	9	7	3	0	0	2	0
DNA_alkylation	DNA alkylation repair enzyme	(PF08713)	191	169	189	78	91	156	50	43	70	76	26	35	66	9	1	1	1	0
TcdB_toxin_midN	Insecticide toxin TcdB middle/N-terminal region	(PF12256)	13	42	38	18	10	20	11	6	3	5	3	4	3	0	1	0	1	0
TnpB_IS66	IS66 Orf2 like protein	(PF05717)	190	224	148	244	227	166	45	123	91	62	30	73	9	18	0	1	21	0
Cas_Cas6	CRISPR associated protein Cas6, C-terminal	(PF01881)	4	5	4	7	13	4	34	21	13	41	93	8	54	292	108	342	71	203
PTR2	Proton-dependent oligopeptide transporter	(PF00854)	86	75	21	67	78	71	76	56	64	77	40	39	15	13	6	0	12	0
ATP-grasp_2	ATP-grasp domain	(PF08442)	269	311	350	375	342	317	333	312	367	332	375	210	418	402	475	570	687	395
BAAT_C	BAAT / Acyl-CoA thioester hydrolase C terminal	(PF08840)	18	9	10	7	10	9	3	6	2	5	1	1	0	0	0	1	2	0
SspB	Stringent starvation protein B	(PF04386)	56	58	8	41	41	49	41	44	72	41	34	28	3	5	0	0	7	0
Rod-binding	Rod binding protein	(PF10135)	15	12	7	17	17	30	22	28	23	17	12	7	6	6	2	0	5	0
5_nucleotid	5' nucleotidase family	(PF05761)	32	27	20	22	25	40	2	1	12	2	0	13	4	0	0	0	0	0
Cu-binding_MopE	Putative metal-binding motif	(PF11617)	78	82	123	56	76	65	45	18	38	26	10	20	3	5	0	0	0	0
Carb_anhydrase	Carbonic anhydrase	(PF00194)	27	23	10	17	8	17	9	0	2	1	5	5	0	1	0	0	8	0
RHS_repeat	RHS Repeat	(PF05593)	489	561	800	684	536	620	185	620	289	388	225	314	363	343	55	307	283	8
Glyoxalase_2	Glyoxalase-like domain	(PF12681)	320	254	130	254	195	439	100	140	268	145	84	277	45	54	62	17	22	0
Fic	Fic/DOC protein family	(PF02661)	352	409	228	158	168	230	123	95	120	102	64	128	66	27	2	33	8	6
Glutaminase	Glutaminase	(PF04960)	113	135	79	109	74	140	68	64	25	52	20	294	6	7	0	0	22	0
Lectin_C	C-type lectin	(PF00059)	14	10	14	11	9	11	3	3	5	0	5	6	2	2	1	10	0	0
Usg	Usg-like family	(PF06233)	45	25	4	25	29	44	19	18	40	11	13	16	3	1	0	0	1	0
TauD	TauD protein domain	(PF02668)	119	138	38	209	94	187	47	46	257	17	75	78	7	13	0	0	9	0
SHD1	SLA1 homology domain 1, SHD1	(PF03983)	16	14	9	4	6	19	2	0	1	0	1	0	0	0	0	0	0	0
ORF6N	ORF6N domain	(PF10543)	106	82	147	42	14	25	11	21	50	15	11	7	0	0	0	0	0	0
PilZ	PilZ domain	(PF07238)	64	49	181	75	80	65	45	48	48	36	45	50	38	15	17	27	25	24
LZ_Tnp_IS66	Transposase C of IS166 homeodomain	(PF13007)	66	54	23	24	19	40	26	29	41	20	8	17	2	4	0	0	15	0
PHB_acc	PHB accumulation regulatory domain	(PF05233)	27	24	3	21	33	23	23	32	50	16	12	7	2	0	0	0	0	0
Ceramidase_alk	Neutral/alkaline non-lysosomal ceramidase, N-terminal	(PF04734)	68	32	15	15	19	15	8	9	4	6	4	7	18	4	0	0	0	6
Autotrns_rpt	Passenger-associated-transport-repeat	(PF12951)	73	115	66	58	103	169	49	29	42	45	59	25	29	22	2	0	0	0

EthD	EthD domain	(PF07110)	25	42	32	33	53	28	6	21	23	17	6	10	5	15	3	37	57	0
ROS_MUCR	ROS/MUCR transcriptional regulator protein	(PF05443)	89	61	21	50	52	78	22	37	69	107	24	19	5	10	1	0	0	0
ATP12	ATP12 chaperone protein	(PF07542)	4	13	3	21	21	9	6	17	31	9	11	13	1	0	0	0	0	0
CstA	Carbon starvation protein CstA	(PF02554)	177	204	294	205	234	144	250	239	224	225	242	46	348	447	595	839	1062	845
FxsA	FxsA cytoplasmic membrane protein	(PF04186)	37	50	5	28	14	42	10	9	21	7	6	15	0	4	0	0	1	0
HTH_Tnp_IS66	zinc-finger binding domain of transposase IS66	(PF13005)	75	70	74	75	73	55	29	66	32	38	14	15	19	4	0	0	21	1
AraC_E_bind	GyrI-like small molecule binding domain	(PF06445)	72	64	91	58	14	144	26	8	33	10	22	14	3	2	0	0	1	0
GATase	Glutamine amidotransferase	(PF00117)	1120	1120	1038	1119	1235	1155	1342	1265	1289	1290	1423	1013	1418	1496	1484	1898	2306	1894
MT-A70	MT-A70	(PF05063)	38	23	29	35	22	32	12	15	66	12	23	20	4	0	4	0	1	0
Tim44	Tim44-like domain	(PF04280)	54	43	35	59	36	67	32	22	82	17	38	21	5	5	1	0	6	0
Erythro_esteras	Erythromycin esterase	(PF05139)	59	184	23	157	40	124	23	32	62	20	19	26	2	7	0	0	0	0
Phage_base_V	Type VI secretion system/phage-baseplate injector OB domain	(PF04717)	43	48	23	46	57	53	24	24	29	20	8	17	59	30	0	52	18	2
Glyco_hydro_6	Glycoside hydrolase family 6	(PF01341)	11	15	2	12	7	12	1	0	0	0	1	1	0	0	0	0	0	0
CmcH_NodU	Carbamoyltransferase N-terminus	(PF02543)	352	404	311	375	394	264	213	260	313	137	203	93	167	316	10	71	162	1
Cu2_monoox_C	Copper type II ascorbate-dependent monooxygenase	(PF03712)	39	47	8	41	50	42	6	29	38	9	17	16	16	17	2	31	8	0
CobT_C	Cobalamin biosynthesis protein CobT VWA domain	(PF11775)	72	63	5	48	81	54	23	41	74	43	16	28	6	4	0	0	0	0
FBPase_2	Fructose 1,6-bisphosphatase	(PF06874)	42	41	21	19	10	32	3	10	5	10	5	11	0	0	0	1	0	0
Glycolytic	Fructose-bisphosphate aldolase	(PF00274)	130	200	52	152	94	214	28	43	168	30	59	42	13	0	0	1	0	0
PG_binding_1	Peptidoglycan binding domain	(PF01471)	148	184	94	254	175	325	132	134	164	82	105	976	26	54	43	10	14	0
Stig1	Stigma-specific protein, Stig1	(PF04885)	7	7	6	12	9	24	5	13	7	8	3	4	0	0	0	0	0	0
Z1	Z1 domain	(PF10593)	36	39	13	16	22	34	31	5	12	4	3	5	1	0	0	0	1	0
