# Supplementary material for: Microbial Functional Diversity Correlates with Species Diversity along a Temperature Gradient
Source: mSystems. 2022 Feb 15;7(1):e00991-21. doi: 10.1128/msystems.00991-21 (PMC8845567; doi:10.1128/msystems.00991-21)
Supplement: TABLE S4 [file msystems.00991-21-st004.pdf]

Sample	Temp (°C)	Total Reads (F + R)	Reads with validated Pfam	% Reads with validated Pfam
**FS1**	21.2	2,660,450	538,782	20.3
**FS5**	24.0	6,999,658	923,081	13.2
**D1**	30.0	10,330,470	2,048,124	19.8
**LN4**	33.1	16,457,856	2,993,587	18.2
**D37**	36.9	5,173,070	843,774	16.3
**RC4**	37.9	3,840,888	753,399	19.6
**N4**	45.0	19,154,944	4,382,751	22.9
**D47**	46.5	5,824,428	897,209	15.4
**LN5**	50.4	26,554,712	5,967,541	22.5
**D54**	54.0	5,260,764	827,757	15.7
**KP3**	54.0	12,313,914	2,735,908	22.2
**GH3**	57.2	6,182,300	1,305,945	21.1
**DC8**	62.8	16,110,352	2,992,237	18.6
**DC2**	66.4	15,015,840	2,912,662	19.4
**HS1**	67.0	7,037,502	992,349	14.1
**DC6**	77.4	7,842,250	2,082,427	26.6
**DC5**	85.9	9,873,004	1,993,092	20.2
**N89**	88.8	5,593,028	724,735	13.0
